# Oligocene niche shift, Miocene diversification – cold tolerance and accelerated speciation rates in the St. John’s Worts (*Hypericum*, Hypericaceae)

**DOI:** 10.1186/s12862-015-0359-4

**Published:** 2015-05-06

**Authors:** Nicolai M Nürk, Simon Uribe-Convers, Berit Gehrke, David C Tank, Frank R Blattner

**Affiliations:** Centre for Organismal Studies Heidelberg, University of Heidelberg, Im Neuenheimer Feld 345, 69120 Heidelberg, Germany; Department of Biological Sciences, Institute for Bioinformatics & Evolutionary Studies, University of Idaho, 875 Perimeter MS 3051, Moscow, ID 83844-3051 USA; Institute of Special Botany, Johannes Gutenberg University, Anselm-Franz-von-Bentzelweg 9a, 55099 Mainz, Germany; Institute of Plant Genetics and Crop Research (IPK), Correnzstrasse 3, 06466 Gatersleben, Germany; German Centre of Integrative Biodiversity Research (iDiv) Halle-Jena-Leipzig, Deutscher Platz 5e, 04103 Leipzig, Germany

**Keywords:** Adaptive landscape, BAMM, Bayou, Divergence time estimation, Climate change, Cold tolerance, Diversification rate shifts, Historical biogeography, *Hypericum* (St. John’s wort, Hypericaceae), Niche shift

## Abstract

**Background:**

Our aim is to understand the evolution of species-rich plant groups that shifted from tropical into cold/temperate biomes. It is well known that climate affects evolutionary processes, such as how fast species diversify, species range shifts, and species distributions. Many plant lineages may have gone extinct in the Northern Hemisphere due to Late Eocene climate cooling, while some tropical lineages may have adapted to temperate conditions and radiated; the hyper-diverse and geographically widespread genus *Hypericum* is one of these.

**Results:**

To investigate the effect of macroecological niche shifts on evolutionary success we combine historical biogeography with analyses of diversification dynamics and climatic niche shifts in a phylogenetic framework. *Hypericum* evolved cold tolerance *c.* 30 million years ago, and successfully colonized all ice-free continents, where today ~500 species exist. The other members of Hypericaceae stayed in their tropical habitats and evolved into ~120 species. We identified a 15–20 million year lag between the initial change in temperature preference in *Hypericum* and subsequent diversification rate shifts in the Miocene.

**Conclusions:**

Contrary to the dramatic niche shift early in the evolution of *Hypericum* most extant species occur in temperate climates including high elevations in the tropics. These cold/temperate niches are a distinctive characteristic of *Hypericum*. We conclude that the initial release from an evolutionary constraint (from tropical to temperate climates) is an important novelty in *Hypericum*. However, the initial shift in the adaptive landscape into colder climates appears to be a precondition, and may not be directly related to increased diversification rates. Instead, subsequent events of mountain formation and further climate cooling may better explain distribution patterns and species-richness in *Hypericum*. These findings exemplify important macroevolutionary patterns of plant diversification during large-scale global climate change.

**Electronic supplementary material:**

The online version of this article (doi:10.1186/s12862-015-0359-4) contains supplementary material, which is available to authorized users.

## Background

At the onset of evolutionary theory it was observed that “species of the same genus have usually […] some similarity in habits and constitution” ([1], p 76). That is, closely related species or lineages are expected to be more similar in ecology than to more distantly related taxa because of their more recent common ancestry [2,3]. Accordingly, it has been observed that under changing environmental conditions organisms tend to retain their ancestral ecological characteristics rather than evolving into a new ecological niche [4-6].

Large-scale global climate change dramatically alters the distribution of major biomes [7], and thus the ecological niches available to entire taxonomic groups [8,9]. During the Early Eocene Climatic Optimum, *c*. 55 million years (Ma) ago, tropical biomes dominated the Earth’s surface even at high latitudes [10]. Within the last 50 Ma, however, the world has experienced a transition: a fluctuating but overall decrease in mean temperatures [7,11,12] that shifted the distribution of frost-intolerant plants towards the equatorial zones. The flowering plant families Araceae [13], Chloranthaceae [14], and Malpighiaceae [15] are well-studied examples of formerly more widely distributed lineages that are currently mostly restricted to the tropics.

Only some lineages of flowering plants have managed the transition from tropical to temperate climates [16,17], despite presumably having had ample opportunities to do so with the Neogene expansion of temperate habitats [18,19]. Adaptation to cold is supposed to involve complex reorganizations of the genome and physiology [20], implying that the evolution of tolerances to temperate climates with highly seasonal conditions may pose particular problems [21,22], especially for warm-adapted plant taxa confronted with cooling climate [19]. In contrast, all extant plant lineages that do occur in temperate areas underwent adaptation to cooler climates at some point [23,24]. Also, in an area that undergoes environmental changes and that lacks suitable migration routes, only such resident lineages will survive that can develop the relevant traits necessary to persist [25-27]. In a recent study of angiosperms, Zanne et al. suggest that “many of these solutions were probably acquired before their foray into the cold” [24]. On the other hand, it has been suggested that in the absence of obvious key innovations, climate cooling may have acted as an important driver of diversification, *e.g.*, in the hyper-diverse genus *Carex* [28].

**Figure 1:**
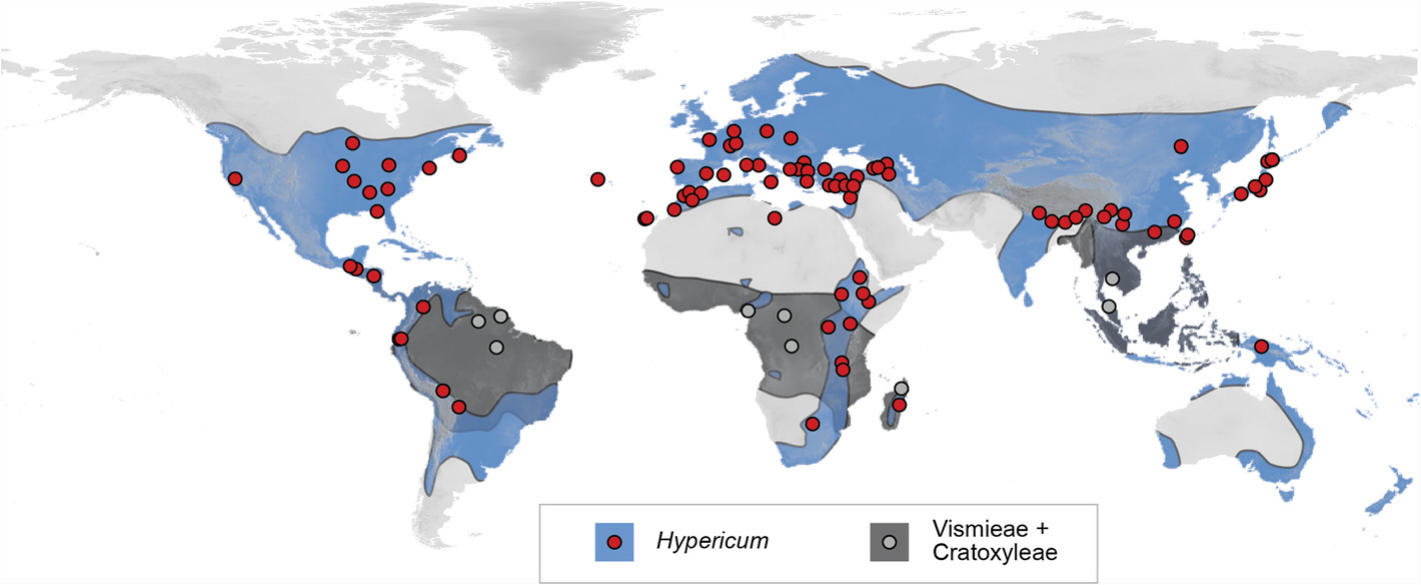
Distribution map of Hypericaceae and collection sites. Blue shading and collection sites (points) in red mark the distribution of *Hypericum* (Hypericeae). Grey shading and collection sites in light-grey mark the distribution of the tropical members of the family (Cratoxyleae and Vismieae).

We provide a macroevolutionary study of the genus *Hypericum* L. (St. John’s Worts, Hypericaceae Juss.) in the context of changing global climate conditions during the Neogene. *Hypericum* belongs to the clusioid clade of the Malpighiales [29,30], which, apart from *Hypericum*, is almost exclusively composed of tropical taxa (Figure 1). More than 80% of the known species within the family Hypericaceae occur within *Hypericum* [31,32]; the remaining 20% consist of tropical plants from four genera [33] in the tribes Vismieae Choisy (*Vismia* Vand., and *Harungana* Lam., incl. *Psorospermum* Spach) and Cratoxyleae Benth. & Hook.f. (*Cratoxylum* Blume and *Eliea* Cambess*.* [34]).

*Hypericum* has a nearly cosmopolitan distribution (Figure 1) with a primary center of species-richness in the temperate regions of Eurasia [35,36]. In temperate regions, *Hypericum* species are mostly native to low- and mid-elevation areas, while in the tropics they are almost always confined to high-elevation mountains, such as the South American Andes or mountains in topical Africa [37]*.* The divergence time of the *Hypericum* crown node has been estimated to the Upper Eocene, *c.* 35 Ma ago [38], based on a single (seed-) fossil calibration. The same study reconstructed the Western Palearctic as the ancestral area for *Hypericum*. However, Sánchez Meseguer et al. [38] employed a Bayesian approach [39,40] that limits ancestral areas to only single states in the reconstruction (*i.e.* it is not possible to infer the occurrence of ancestral populations in multiple areas). For a genus like *Hypericum* that has several species that span very wide geographic ranges, this assumption is likely inadequate.

Within an otherwise pantropic clade [30] *Hypericum* is a cold-adapted but species-rich lineage. Because the clade is of worldwide cold/temperate distribution and evolved during the Paleaogene to Neogene (23.3 Ma [41]) climate transition, *Hypericum* is ideal for studying the effects of broad-scale climate change on plant distribution and diversification. Furthermore, the evolution of morphological characters in the genus has been studied at length [36-38,42], providing a foundation to further investigate evolutionary patters and processes in *Hypericum*.

In this study, we test the hypotheses that (*i*) *Hypericum* originated in the Western Palearctic prior to the Oligocene, as suggested by Meseguer et al. [38], that (*ii*) the occupation of temperate environments is derived in the Hypericaceae and a distinctive characteristic of *Hypericum*, and that (*iii*) it has stimulated the radiation of this lineage globally. To do so, we estimate a species phylogeny based on nuclear and chloroplast sequence variation. We calibrate our time-tree using six fossils and compare the effect on node ages of the specific assignment of the oldest known fossil in *Hypericum*. To evaluate historical biogeography, we employ parametric models that allow for the incorporation of paleogeographic information. Applying recently developed Bayesian approaches, we estimate the magnitude and placement of climatic niche shifts, and investigate the impact of the transition from the tropical into the cold/temperate climate niche by assessing the placement of shifts in diversification rates.

## Results

### Hypericaceae phylogenetics

Maximum likelihood (ML) topologies were similar for both chloroplast (*pet*D + *trn*L–*trn*F) and nuclear (ITS) data. Discordance between nuclear and chloroplast trees was present only in two places, although without strong support (Additional file 1: Phylogenetic inference). Thus, we concatenated the chloroplast and nuclear sequence data into a combined data set, which contained 24.1% missing data (0.1% missing in ITS, 21.2% in *pet*D, and 59.4% in *trn*L–*trn*F).

**Figure 2:**
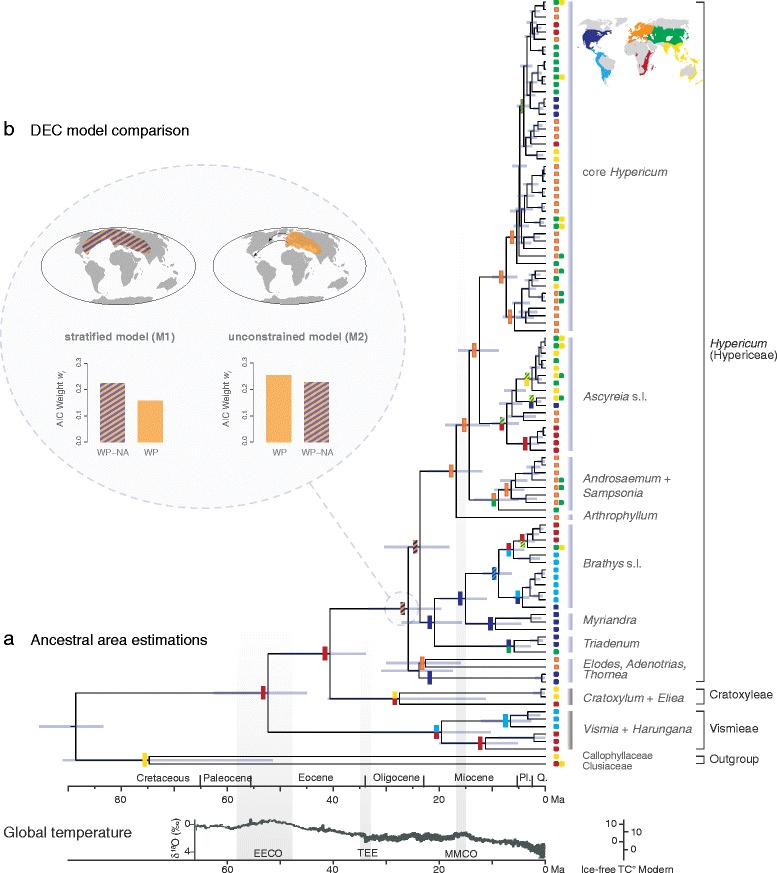
Dated phylogeny of Hypericaceae detailing historical biogeography. **(a)** Present occurrence of species is marked at the tips of the tree using the color code defined in the map top right. Multiple occurrences are indicated. Historical distribution of ancestral populations is given at nodes in the tree (ancestral areas estimated under the M1 model). Node bars indicate the 95% highest posterior density (HPD) produced in divergence time estimation A. Vertical bars define the clades used to assign species-richness in the diversification rates analysis. **(b)** Comparison of ancestral areas optimized for the *Hypericum* crown node under two DEC models, the stratified (M1) and the uncostrained (M2). Maps illustrate the reconstructed distribution of ancestral populations and bar charts the likelihood of range optimization (expressed by AIC weights *w*_*i*_; WP, western Palearctic; NA, North America). Global temperature (oxygen-isotope curve as a proxy for temperature [11]) is given below the geological time scale. Grey vertical bars indicate major climatic events (EECO, Early-Eocene Climatic Optimum; TEE, Terminal Eocene Event; MMCO, Mid-Miocene Climatic Optimum; Ma, million years ago). Note that the cold adapted *Hypericum* lineage splits form its tropical sister and starts to diversify during periods of climate cooling.

**Figure 3:**
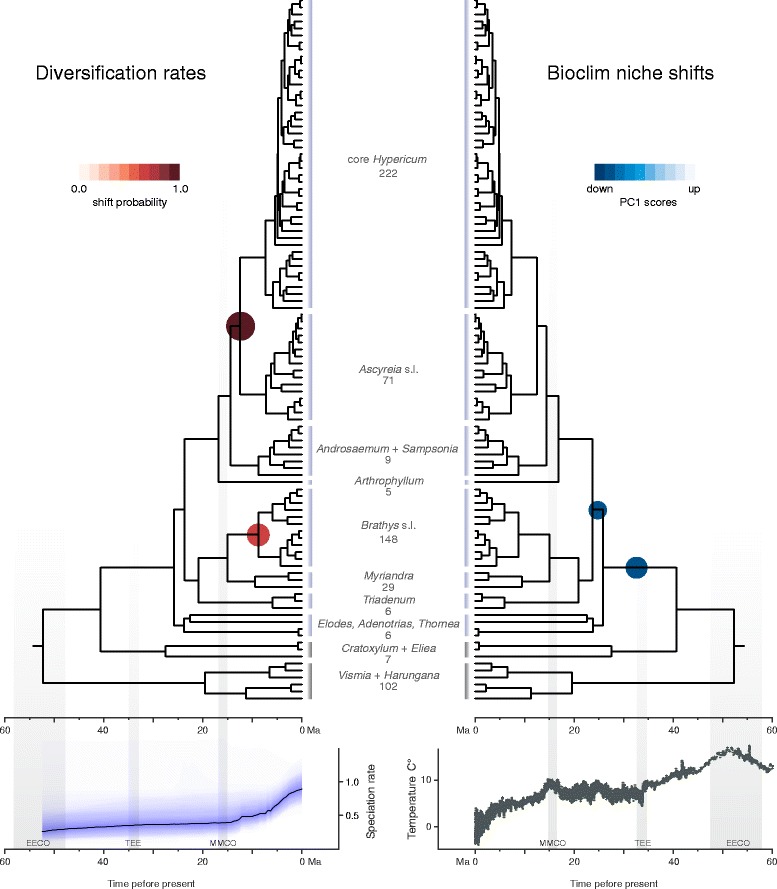
Results of diversification rate and niche analyses. Species richness of the clades is given below the clade names. Diversification rate shifts (to the left) are located on the respective nodes in the tree by circles colored according to the scale detailing the marginal shift probability. A rate through time (RTT) plot is given below the tree detailing speciation rates, blue-shaded polygons denote the 10% through 90% Bayesian credible regions on the distribution of rates. Shifts in the adaptive landscape to a new climatic niche are located on the respective branches in the tree (to the right) by circles colored according to the scale detailing the new phenotypic optima (in PC1 score units). Note that shifts in the climatic niche coincide with the Oligocene climate cooling shown in the global temperature (oxygen-isotope) curve [11] below (temperature scale adjusted to modern ‘glaciated Antarctica’ situation following [7]). Grey-shaded bars denote the Early-Eocene Climatic Optimum (EECO), the Terminal Eocene Event (TEE), and the Mid-Miocene Climatic Optimum (MMCO).

We included 100 representative species (103 accessions; online Additional file 1: Voucher) in the combined sequence data set that contained 2024 nucleotide positions after alignment and removal of ambiguous sites. Phylogenetic inference revealed the same major groups as reported in other studies [37,38,43,44], but with strong support (Additional file 1: Figure S1). Most basal splits within *Hypericum*, however, lack sufficient support, a result also revealed in previous studies that included deeper sampling [37,38]. Ten major clades consistent with current taxonomy are present within Hypericaceae (Figure 2 and Additional file 1: Figure S1), allowing us to assign species richness to each clade for comparative analyses (Figure 3).

### Divergence times depend on fossil assignment

Bayesian estimation of divergence times was done by assigning the oldest known fossil in *Hypericum* as a minimum time constraint to (A) the stem node, and (B) to the crown node of *Hypericum* (Additional file 1: Calibration; Additional file 1: Figure S2). For both analyses, we tested the effect of missing data on age estimates using a reduced data set that did not contain any missing data. Results of the complete sampling and the reduced data sets were congruent, regardless of the calibration approach used, deviating maximally at the *Hypericum* node with less than 1.6 Ma in mean ages (Table 1).

**Table 1:** **Results of divergence time estimation using different fossil assignments**

**Node (mrca)**	**Bayesian relaxed clock – crown ages**			
	**Analysis A**		**Analysis B**	
	**complete sampling**	**‘no missing’ data**	**complete sampling**	**‘no missing’ data**
Hypericaceae	52.31	53.13	59.63	57.22
	(62.66–45.00)	(64.05–45.15)	(71.25–49.26)	(69.06–47.94)
Vismieae	19.59	—	23.25	—
	(32.66–10.22)		(37.69–12.73)	
Cratoxyleae	27.52	—	32.50	—
	(41.08–11.23)		(48.89–14.38)	
Hypericum	25.87	27.45	35.20	35.77
	(33.32–19.59)	(36.50–19.00)	(39.31–33.90)	(40.97–33.90)
Brathys s.l. + Myriandra	15.03	16.12	19.63	20.70
	(19.99–10.99)	(22.67–10.77)	(24.56–14.87)	(26.52–15.01)
Brathys s.l.	8.77	8.97	11.08	11.24
	(11.82–6.26)	(13.06–5.76)	(14.63–7.94)	(15.28–7.71)
core Hypericum + Ascyreia s.l.	12.46	12.65	15.86	15.54
	(16.44–8.80)	(18.00–8.40)	(20.63–11.66)	(21.17–10.80)

Mean crown ages in Ma are given with the 95% HPD in brackets below. HPD, highest posterior density; Ma, million years.

Both calibration approaches (A and B) are detailed, and results of analyses A and B repeated using the reduced data set that did not contain missing sequence data (‘no missing’ data).

Divergence time estimates produced by the two analyses (A and B) differed by ~10 million years at the *Hypericum* crown node (Table 1 and Additional file 1: Table S1). Analysis A is congruent with Xi et al. [30], which focused on family level relationships and estimated divergence times within Malpighiales using 82 chloroplast DNA regions and 16 external fossil calibrations. In analysis B the inferred crown age is *c.* 8 million years older for the Hypericaceae (the most recent common ancestor [MRCA] of *Hypericum*, Vismieae, and Cratoxyleae; Table 1 and Additional file 1: Table S1) compared to Xi et al. [30]. It is conservative to assign a fossil to a stem node given that a hard minimum time constraint is used for calibration [45]. Therefore, we use the divergence time estimate of analysis A (fossil assignment to the stem node; mean crown age of *Hypericum* 25.9 Ma [33.3–19.6 95% HPD]; Table 2, Additional file 1: Figure S2) to discuss the results of the biogeographic optimization, diversification analyses, and climatic niche shifts in the historic context (results of these analyses using both divergence time estimations are given in the supplementary materials; Additional file 1: Table S1, S2, and S3, Figures S4 and S5).

**Table 2:** **Summary statistics detailing node support, age estimation, diversification rates, and niche shifts**

**Node (mrca)**	**Node support**	Crown age*	Diversification rates*		Bioclimatic niche shifts*	
	**(pp|ML)**	**(Ma)**	**shift probability**	**speciation rate**	**pp**	**phenotypic optimum**
Hypericaceae	1|100	52.31	—	0.66	—	0.00
		(62.66–45.00)		(0.46–0.88)		
Vismieae	1|100	19.59	—	0.43	—	1.63
		(32.66–10.22)		(0.19–0.83)		(0.07)
Cratoxyleae	.56|60	27.52	—	0.41	—	1.11
		(41.08–11.23)		(0.18–0.81)		(0.15)
Hypericum	1|100	25.87	—	0.75	0.53	−4.24
		(33.32–19.59)		(0.54–1.0)		(0.01)
core Hypericum – Brathys s.l.	.83|65	23.67	—	0.83	0.34	−4.40
		(30.41–18.08)		(0.59–1.08)		(0.01)
Brathys s.l.	1|100	8.77	0.64	1.04	—	−3.67
		(11.82–6.26)		(0.54–1.75)		(0.06)
core Hypericum + Ascyreia s.l.	1|99	12.46	0.93	1.06	—	−4.67
		(16.44–8.80)		(0.75–1.51)		(0.03)

*Results produced using age estimation A. For the diversification rate analysis, detected shifts are marked by their probability. The mean speciation rate (species/Ma) per clade is detailed with the 5%, and 95% HPD in brackets below. For the bioclimatic niche analysis, shifts are marked by their probability, and the new phenotypic optimum (PC1 score optimum) is detailed with the standard error in brackets below. HPD, highest posterior density; pp, posterior probability; ML, maximum likelihood bootstrap support; Ma, million years; shift probability, marginal probability of rate shifts.

### Ancestral area estimation is equivocal at the *Hypericum* crown node

Ancestral areas were optimized over both divergence time estimations taking phylogenetic uncertainty into account by analyzing a posterior subset of 1000 trees. Additionally, two models were compared which differed in their dispersal/extinction probabilities both between areas and over time; a stratified M1 model that accounts for varying connectivity of areas over time (Additional file 1: Figure S3), and a unconstrained M2 model with equal probabilities of movement between areas at any time (*i.e.* equal dispersal/extinction probabilities). The ancestral areas estimated per node were highly congruent in all analyses, except for four nodes for which the ancestral states differed (Additional file 1: Table S2). Two of these affected nodes are located at basal dichotomies, one at the *Hypericum* crown node (Figure 2), and one at the ‘*Ascyreia* s.l.’ crown node. In all four cases of incongruence, and independent of the used age estimation, the evidence ratio [46] is generally higher in the stratified model when compared to unconstrained model (Additional file 1: Table S2).

### Niche shifts during the evolution of the *Hypericum* stem lineage in the Oligocene

We used the first principal components (PC1) scores obtained from a phylogenetic PCA as an approximation of the climatic niche. The PC1 scores were dominated by the thermal bioclim variables (bio1–11; with the most influence from mean annual temperature [bio1]), and account for *c.* 34% of the variation within the data. Bayesian fitting of multi-optima OU models to the approximation of the climatic niche space estimated the placement and magnitude of two shifts. Both shifts were estimated to have occurred in the Oligocene, the first shift during the evolution of the stem lineage of *Hypericum*, and the second shift one divergence event latter at the stem lineage of the MRCA of ‘core *Hypericum’* – ‘*Triadenum* (Table 2; for a comparison of results produced under the two age estimations see Additional file 1: Figure S5, Table S3). Both shift magnitudes were negative, indicating a niche shift into colder climates during the early evolution of *Hypericum* (Table 2 and Additional file 1: Table S3). The phylogenetic half-life (ln(2)/α = 0.009 Ma; based on a rate of adaptation of α = 1.525, and a total tree length of 52.31 Ma) signifies that the movement to the primary climatic optimum in *Hypericum* was rapid and resembles an OU process rather than Brownian motion [47].

### Diversification rate shifts in *Hypericum* in the Miocene

In the Hypericaceae, two shifts in diversification dynamics were detected within *Hypericum* with strong support: (*i*) at the stem lineage of ‘core *Hypericum + Ascyreia* s.l.’, and (*ii*) with the divergence of Central- and South American ‘*Brathys* s.l.’ from its relatives (Figure 3, Table 2). In both dating approaches, the same clades are identified to show diversification rate shifts comparing the Bayesian credible sets of distinct shift configurations (Additional file 1: Figure S4). In the analysis using the dating approach A, the first increase in diversification rates is inferred to have occurred at about 13.93 Ma with a marginal probability of 0.93, and the second at about 12.54 Ma with a marginal probability of 0.64, resulting in a rate of 1.04 and 1.06 species per million year, respectively (for a comparison of results see Additional file 1: Figure S4, Table S3).

## Discussion

### Historical biogeography of Hypericaceae

There is strong evidence that the MRCA of Hypericaceae occurred in Africa in the Eocene (Figure 2), a result also recovered in a previous study [38]. The MRCAs of the tropical taxa within the Hypericaceae (Cratoxyleae and Vismieae) are revealed to have occurred in Africa + Southeastern Asia and Africa + South America, respectively. During the Early Eocene the higher thermal maximum allowed megathermal organisms, such as the MRCA of Hypericaceae, to disperse throughout the Northern Hemisphere, which explains the vicariance of many tropical lineages [10,19,48].

At the onset of worldwide climate cooling, *c.* 40 Ma the *Hypericum* stem lineage split from its tropical relatives. The ancestral area estimated for the MRCA of *Hypericum* is in the Northern Hemisphere, either solely in the Western Palaearctic, or more widely distributed between the Western Palaearctic and the Nearctic (Figure 2, Additional file 1: Table S2). The Nearctic has been connected to the Western Palearctic through islands that could have easily functioned as stepping-stones facilitating dispersal over the North Atlantic [49-51]. Seeds of *Hypericum* are tiny, ca. 1.5 to 2.5 mm long, and are easily dispersed by birds or strong winds [32], promoting long-distance dispersal. However, an ancestral distribution from the Western Palaearctic to the Nearctic is somewhat surprising given the fact that the oldest known fossil is from Siberia and SW China [52]. Differential extinction may explain the partial absence of reconstructed ancestral populations in the areas of the oldest fossil record [38]. Hence, the assumption of a widespread MRCA of *Hypericum* from the Palaearctic to the Nearctic connected via Beringia [19,53] would be plausible as well. On the other hand, it is likely that the fossil record does not accurately represent actual ancestral distributions, and thus, may be misleading for ancestral area estimations. Regardless, *Hypericum* is of Northern Hemisphere origin, likely around the late Tethys estuaries in the Palaearctic [37], perhaps as part of a deciduous mixed-mesophytic forest [54]. The ecology of basal lineages within *Hypericum*, however, is diverse reaching from dry rocky Mediterranean to shallow aquatic habitats, and montane cloud forests. Thus, extensive differential extinction of intermediate ancestral populations (ecologically and geographically) and/or intercontinental long-distance colonization early in the evolutionary history is needed to explain biogeographic patterns in *Hypericum.*

### Cold adaptation in the *Hypericum* stem lineage but later diversification

Initially, no speciation seems to have taken place in *Hypericum* – or extinction may have erased the evidence of earlier divergence in the genus leading to a stem lineage of *c.* 15 million years (*c.* 40–25 Ma). However, the genesis of modern species diversity in *Hypericum* traces to *c.* 25 Ma (Figures 2 and Additional file 1: Figure S2), after the Oligocene climate cooling caused a substantial decrease in mean temperatures [11]. This worldwide cooling, which led to a period of rather constant cold temperatures [55], initiated the expansion of temperate habitats in the Northern Hemisphere, replacing the more tropical vegetation dominant during the Eocene [10]. With the exception of *Hypericum*, the remaining lineages of Hypericaceae stayed in tropical climates, *i.e.* their distributions experienced a restriction with the retreat of tropical areas towards equatorial zones after the Eocene Thermal Maximum [10]. In contrast, *Hypericum* adapted to colder climates *c.* 30 Ma (Figure 3) and consequently dispersed and diversified in temperate habitats. The adaptation towards the new climatic optimum (estimated by the phylogenetic half-life [56]) was rapid, meaning that *Hypericum* species were well adapted to the cold/temperate niche [47]. Hence, following this initial niche shift, *Hypericum* never completely left the colder climates. Even in equatorial areas, *Hypericum* is only found at high elevations with a cool climate, such as the South American Andes and the high mountains of Africa.

Two shifts in diversification rates were detected with strong support in *Hypericum* (Figure 3). The first is an increase in speciation rates that coincides with a sudden decrease of temperature during the Middle Miocene Climate Transition at about 14 Ma [11]. The second increase in speciation rates was likely the result of dispersal into the South American continent and may have been triggered by the orogeny of the Andes [43]. That is, the adaptation of *Hypericum* to colder climates first evolved during the Oligocene but diversification rates increased *c.* 13–17 million years later during the Miocene, in nested clades within *Hypericum*. The evolution of a shrubby habit at the *Hypericum* stem lineage [37] might well be a life history trait that facilitated the initial adaptation to cold [24], but no obvious intrinsic (morphological or physiological) traits that could be interpreted as key innovations were identified for these rapidly diversifying clades [37,38]. However, our results suggest that cold tolerance is likely an important initial adaptation that was exploited when new biogeographic opportunities were presented (*i.e.* Andean orogeny). Only after a long lag phase when global temperatures dramatically decreased following the Mid-Miocene Thermal Optimum [11], did changing environments and expanding temperate regions (including tropical high mountains) allow *Hypericum* to spread and diversify. Because *c.* 42% of extant *Hypericum* species are found in montane biomes (*c.* 72% of this diversity in tropical high mountains), we postulate that the onset of extensive mountain formation in Eurasia and the Americas [57] is likely to have contributed to both detected rate shifts.

## Conclusion

After divergence from its tropical relatives, adaptation towards colder climates *c*. 30 Ma ago enabled *Hypericum* to stay in the Northern Hemisphere, while its tropical relatives experienced habitat restriction towards equatorial zones. This niche shift offered dispersal and possible diversification opportunities in the expanding temperate areas in the Northern Hemisphere during the Oligocene. After the last thermal maximum in the Miocene, massive mountain formations and further climate cooling may have stimulated the radiation of this lineage globally. As a consequence, cold-adapted *Hypericum* contains 80% of the species present in the family. Despite its worldwide distribution and tropical ancestry, even the species growing in the tropics retain their temperate climate niche by growing exclusively in cool climates of higher elevation habitats.

Higher species richness in temperate climates is rather atypical and contrary to the usual pattern observed in plants where tropical groups generally tend to have more species than temperate relatives [6,23]. However, our findings mirror patterns described in sedges (*Carex*) [28], and to varying extents in buttercups (Ranunculaceae) [58], grasses (Poaceae) [59-62], and heaths (Ericales) [63].

We have demonstrated that a pronounced lag phase is present between the initial niche shift and diversification in new habitats. Therefore, we conclude that the disproportionate species numbers of *Hypericum* in comparison to its tropical relatives are not only a result of initial cold-climate adaptation. Our analyses indicate a relatively late increase in speciation rate, *i.e.* with the onset of further cooling and especially mountain formation during the Upper Miocene (and Pliocene). Likewise, *Carex*, the most diverse non-tropical sedge lineage (Cyperaceae) [28] has a stem lineage of *c.* 20 million years, with most extant lineages having diversified subsequent to the Oligocene in montane biomes. Thus, we emphasize that both (*i*) a niche shift following the Eocene Thermal Optimum as a precondition for the presence of cold-adapted lineages in temperate regions, and (*ii*) an extrinsic trait (perhaps in addition to lineage-specific intrinsic traits), *i.e.* the availability of emerging temperate and/or mountain habitats, are key events potentially triggering diversification rates in these cold-adapted, species-rich plant groups.

## Methods

### Taxon sampling and species richness

Plant material from herbarium specimens or silica dried samples were chosen for 100 species (and 3 subspecies) representing the distribution range of all major lineages present in Hypericaceae [30,37] (see Additional file 1: Voucher). Following Xi et al. [30], *Garcinia xanthochymus* Hook.f. ex T.Anderson (Clusiaceae Lindl.) was chosen as outgroup in the phylogenetic analyses.

The monograph of *Hypericum* [31,32,42,64-72] was used for data on species richness and distribution (for *Hypericum sensu* Robson 2012). Stevens [34] lists information for the remaining taxa of Hypericeae Choisy (*Triadenum* Raf., *Thornea* Breedlove & E.M.McClint., and *Lianthus* N.Robson; included in *Hypericum* in Ruhfel et al. [33]), as well as the tropical genera of the family Hypericaceae. Xi et al. [30] provides information on distributions for Clusiaceae and Calophyllaceae J.Agardh (Additional file 1: Voucher). Total species numbers with evidence from taxonomy [32], morphological cladistics [36], and molecular phylogenetic analyses [37,38] were used to assign species richnesses to the major clades defined in the diversification rate analyses.

### Molecular marker and sequencing

We sequenced two fragments from the chloroplast genome, namely *pet*D (including the *pet*B–*pet*D intergenic spacer, the *pet*D-5′-exon, and the *pet*D intron) and *trn*L–*trn*F (including the *trn*L^UAA^ intron and the intergenic spacer between the *trn*L^UAA^ 3′ exon and *trn*F^GAA^ gene), and the nuclear rDNA internal transcribed spacer region (including ITS-1, 5.8S rDNA, and ITS-2). Extraction of DNA was done according to Nürk et al. [37]. In the case of well preserved herbarium material or silica dried samples, entire regions were amplified using the primers ITS-A(F) and ITS-B(R) [73], PI*pet*B1411F and PI*pet*D738R [74], c(F) and f(R) designed by Taberlet [75] for the *trn*L–*trn*F region. In the case of degraded herbarium materials, ITS-1 and ITS-2 were amplified separately using in addition two internal primers, ITS-C(R) and ITS-D(F) binding in the conserved 5.8S rDNA [73]. Similarly for *pet*D, using the two internal primers SAL*pet*D599F and O*pet*D897R designed by Korotkova et al. [76]. PCR amplification of ITS was performed as described in Nürk et al. [37]. PCR reaction mixes for *pet*D and *trn*L–*trn*F were chosen according to Nürk et al. [37], but without adding MgCl_2_, and PCR profiles consisted of an initial denaturation at 96°C for 1.5 min, followed by 35 cycles of 95°C for 30s, 50°C for 60s, 73°C for 90s and a final step at 72°C for 10 min. Primer combinations as described above for poorly preserved samples were used for cycle sequencing. DNA sequencing was done by Eurofins MWG Operon (Ebersberg, Germany). All newly generated sequences have been submitted to the to the EMBL nucleotide database (Accession No. LK871650–LK871782).

### Phylogenetic inference

Sequences were assembled and edited with Geneious v5.4 [77], aligned using the automatic selection of an appropriate strategy in Mafft v6.903b [78,79] and manually adjusted using PhyDE v0.996 (available online: http://www.phyde.de). In order to remove poorly aligned or length variable data partitions the alignments were subjected to Gblocks 0.91b sever [80] with the ‘less stringent’ options selected.

Phylogenetic analyses were performed under maximum likelihood (ML) [81] and Bayesian inference (BI) [82] to reveal confidence limits of the data. ML analyses were performed with the RAxML GUI v1.1 [83,84] and BI in MrBayes 3.2.2 [85]. To test for discordance we analyzed the nuclear (ITS) and chloroplast data partitions (*pet*D, *trn*L–*trn*F) separately by ML search under the GTRCAT model of sequence evolution. Clade support was evaluated with 1000 rapid bootstrap replicates [86].

The combined data set (ITS + *pet*D + *trn*L–*trn*F) was analyzed under ML with the partitions defined and the settings chosen as described for the ML analysis above. For BI we started 4 simultaneous runs, each with 4 chains, set to run 10^8^ cycles, with sampling every 10^4^ cycle, setting temperature to 0.01, and with the appropriate model of sequence evolution specified per partition: GTR + I + Γ for ITS and *pet*D and HKY + I + Γ for t*rn*L–*trn*F; selected in MrModeltest [87] under the Akaike Information Criterion (AIC) [88]. We used the ML tree as a starting tree, but introduced random perturbations into it to enable detection of possible convergence problems (using the command “mcmcp nperts = 5”). A ‘corrected’ exponential prior on a branch length of 1/λ = 0.1 [“prset brlenspr = Unconstrained:Exp(100)”] was specified [89]. Convergence of parameter estimates was monitored using Tracer v1.5 [90]. After discarding 25% of the sampled trees as burnin, posterior probabilities were calculated on the BI stationary sample. Trees and alignments are available at TreeBASE study number 16298.

### Divergence time estimation and fossil assignment

The likelihood ratio test [91] conducted on the BI consensus tree in PAUP* [92] rejected a global molecular clock (*P* < 0.05) for the combined data set. Therefore, divergence times were estimated under a relaxed molecular clock employing the uncorrelated lognormal model [93] in BEAST v1.7 [94]. Eight external time-constraints were imposed for calibration, comprising six fossils [52,95-97] and two secondary calibrations [30] (for details see Additional file 1: Age estimation, calibration). Fossil calibrations were constrained by hard minimum bounds and secondary calibrations by lognormal distributions to incorporate the uncertainty reported in the original study [30]. Two approaches were designed differing only in the assignment of the seed fossil *Hypericum antiguum* Balueva & V.P. Nikitin [97]: (*i*) to the stem node of *Hypericum* in analysis A, and (*ii*) to the crown node of *Hypericum* in analysis B (other calibrations remained unchanged in the two analyses; for a discussion of fossil assignment see Additional file 1: Age estimation, calibration).

A birth and death model of speciation considering incomplete species sampling [98] was set as tree prior. Both divergence time estimations (A and B) were started with two independent Monte Carlo Markov Chain (MCMC) runs, each set to run 10^8^ cycles with sampling every 10^4^ cycles. The substitution and clock models were not linked between the partitions. The ML tree was used as starting tree. To ensure that the prior branching times of the starting tree fulfilled the constraints imposed by the calibration priors we transformed branch length into absolute time using penalized likelihood [99] with the chronopl command in the R [100] package ape [101]. Convergence of parameter estimates was monitored using Tracer [90]. The resulting trees were combined in LogCombiner with a burnin of 50%. On the remaining 10,002 trees means and confidence intervals were calculated in TreeAnnotator [94] to obtain the final consensus tree, the ultrametric time calibrated maximum clade credibility (MCC) chronogram that has 95% of the highest posterior density (HPD). Because missing data can have deleterious effects on analyses that depend on branch length [102], we tested the effect on age estimates of missing sequences in our data set. We repeated both analyses (A and B) using a data set that did not contain missing data (‘no missing’ data) and that had therefore a reduced species sampling containing only 73 accessions (xml input files are available at the Dryad repository [103]).

### Ancestral area estimation

Historical biogeography was analyzed by classifying the species to be distributed within six biogeographic regions, following Brummit et al. [104] for area subdivision. The region were defined to reflect general biogeographic entities, and to be meaningful for the study group: (A) Afrotropical [central Africa, the southern Arabian peninsula, Madagascar, and the West Indian Ocean islands], (WP) western Palearctic, (EP) eastern Palearctic, (IP) Indo-Pacific [SE tropical Asia, Australasia, and the Pacific], (NA) North America [Nearctic], (SA) South America [Neotropic].

We employed a parametric likelihood approach that uses the DEC model [105] implemented in Lagrange [106]. Two models were designed, differing in dispersal probabilities between areas and time to take into account the impact of dispersal/extinction probabilities. The first model (M1) was stratified, *i.e.* incorporating paleogeographic information on area connectivity, *e.g.*, the existence of land bridges, by varying the dispersal probabilities between areas and over time (Additional file 1: historical biogeography; Additional file 1: Figure S3). The second model (M2) was unconstrained, assuming equal dispersal probabilities between areas over time. Ancestral areas were optimized under both models over 1000 post-burnin posterior trees randomly chosen [107] and generated by divergence time estimation A and, in a second analysis, by estimation B. Composite Akaike weights [108] were used to summarize the likelihood of range optimizations. Evidence ratios (the ratio of Akaike weights *w*_*i*_/*w*_*j*_ [46]) were used to evaluate which scenario is the most favored.

### Bioclimatic niche analysis

We used the WorldClim [109] global climate layers considering all 19 Bioclim variables as an approximation of the climatic niche of the species. Collection sites were taken from the voucher specimens, or manually geo-referenced (if not documented on the specimen) while comparing voucher information with collection sites per species recorded in the Global Biodiversity Information Facility (GBIF). The bioclimatic variables for each collection locality were extracted using the R package raster [110]. To identify significant changes in the adaptive landscape (*i.e.* the climatic niche space) within the study group we employed a reversible-jump Bayesian method of fitting multi-optima Ornstein-Uhlenbeck (OU) models to the bioclim variables [47]. To do so, we first transformed the bioclim variables using a phylogenetic principal components analysis (PCA) [111] in the R package phytools [112] to obtain principal components (PC) scores per species while correcting for phylogenetic history (*i.e.* the correlation of independent contrasts [111]). Then, we analyzed the PC1 scores using in the R package bayou [113] with a standard error of 0.5 to estimate the placement and magnitude of shifts in the climatic niche directly from the data [47]. We allowed only one shift per branch and assigned an equal probability of each branch having a shift. We placed a corrected Poisson distribution as prior on adaptive optima and a probability density for a half-Cauchy distribution on the number of shifts between adaptive regimes [using make.prior((tree, dists = list(dalpha = “dhalfcauchy”, dsig2 = “dhalfcauchy”, dsb = “dsb”, dk = “cdpois”, dtheta = “dnorm”), param = list(dalpha = list(scale = 1), dsig2 = list(scale = 1), dk = list(lambda = 15, kmax = 200), dsb = list(bmax = 1, prob = 1), dtheta = list(mean = mean(dat), sd = 2*sd(dat))))], and run MCMC for 10^6^ cycles. To verify that MCMC analyses converged to the same posterior distribution, we applied the Gelman diagnostic [114] in the R package coda [115]. After discarding the first 30% of samples per run as burnin parameter estimates of the two runs were combined and summarized using the Lposterior command in bayou [113] to obtain a posterior of shift locations and magnitudes, the rate of adaptation (α), and phylogenetic half-life (the amount of time it takes for the expected trait value to get halfway to the phenotypic optimum, defined as ln(2)/α units of time [56]).

### Diversification rate analysis

We used the Bayesian approach for studying patterns of rate variation through time and among lineages implemented in BAMM (Bayesian analysis of macroevolutionary mixtures) [116,117]. The method aims at detecting and quantifying heterogeneity in evolutionary rates by using reversible-jump Markov Chain Monte Carlo to detect subclades that share common parameters of speciation and extinction. BAMM identifies sets of shifts (*i.e.* configurations) that are sampled together and allows one to compute relative and marginal probability of those configurations (given in the 95% credible set of distinct shift configurations). We analyzed both the MCC consensus tree produced by divergence time estimation A, and B, respectively (outgroup taxa were removed prior to the analyses to increase statistical power). To do so, we assigned species richness (*i.e.* sampling fraction) to ten well-supported clades to account for incomplete sampling, and ran MCMC over 10^8^ cycles with default settings. Convergence of parameter estimates was evaluated by means of effective samples size (ESS) diagnostics using the R package coda [115] after discarding 10% of samples as burnin. Results were summarized and visualized using the R package BAMMtools [116].

## Additional file

Additional file 1:
**Phylogenetic inference and topological discordance. Figure S1** - Phylogeny of Hypericaceae (detailing species names and node support). Discussion of topological discordance. **Age estimation, calibration.** Discussion of fossil assignment. **Figure S2** - MCC chronogram of Hypericaceae (detailing time-constraints). **Table S1** - Crown group ages of major clades (results of analyses A and B). **Historical biogeography. Figure S3** - Paleogeographical model designed for the stratified ancestral area reconstruction (M1). **Table S2** - Summary statistics obtained by optimization of ancestral areas over 1000 trees generated by age estimations A and B detailing results using models M1 and M2. **Diversification rate analysis. Figure S4** - Diversification rate shifts obtained by analysis A and B, with respective credible sets of distinct shift configurations. **Table S3.** Results of diversification rate analyses under the different age estimations. **Bioclimatic niche analysis.** Niche shifts obtained by analysis A and B. **Table S3**. Results of niche analyses under the different age estimations. **Voucher.** Species in this study, detailing names, reference, EMBL/Genbank ID, and species distribution (area coding and coordinates). The data sets supporting the results of this article are available in the Dryad repository, http://dx.doi.org/10.5061/dryad.4tm8j (BEAST xml input files). Sequence alignments and produced BI/ML trees and are available at the TreeBASE repository http://purl.org/phylo/treebase/phylows/study/TB2:S16298. Original sequence files are stored at the EMBL nucleotide repository, http://www.ebi.ac.uk/ena/data/view/LK871650-LK871782.
