# Supplementary material for: Oligocene niche shift, Miocene diversification – cold tolerance and accelerated speciation rates in the St. John’s Worts (Hypericum, Hypericaceae)
Source: BMC Evol Biol. 2015 May 6;15:80. doi: 10.1186/s12862-015-0359-4 (PMC4422466; doi:10.1186/s12862-015-0359-4)
Supplement: Additional file 1: — Phylogenetic inference and topological discordance. Figure S1 - Phylogeny of Hypericaceae (detailing species names and node support). Discussion of topological discordance. Age estimation, calibration. Discussion of fossil assignment. Figure S2 - MCC chronogram of Hypericaceae (detailing time-constraints). Table S1 - Crown group ages of major clades (results of analyses A and B). Historical biogeography. Figure S3 - Paleogeographical model designed for the stratified ancestral area reconstruction (M1). Table S2 - Summary statistics obtained by optimization of ancestral areas over 1000 trees generated by age estimations A and B detailing results using models M1 and M2. Diversification rate analysis. Figure S4 - Diversification rate shifts obtained by analysis A and B, with respective credible sets of distinct shift configurations. Table S3. Results of diversification rate analyses under the different age estimations. Bioclimatic niche analysis. Niche shifts obtained by analysis A and B. Table S3. Results of niche analyses under the different age estimations. Voucher. Species in this study, detailing names, reference, EMBL/Genbank ID, and species distribution (area coding and coordinates). The data sets supporting the results of this article are available in the Dryad repository, http://dx.doi.org/10.5061/dryad.4tm8j (BEAST xml input files). Sequence alignments and produced BI/ML trees and are available at the TreeBASE repository http://purl.org/phylo/treebase/phylows/study/TB2:S16298. Original sequence files are stored at the EMBL nucleotide repository, http://www.ebi.ac.uk/ena/data/view/LK871650-LK871782. [file 12862_2015_359_MOESM1_ESM.pdf]

BMC evolutionary biology additional file 1

Oligocene niche shift, Miocene diversification – cold tolerance and accelerated speciation rates in the St. John's Worts (*Hypericum*, Hypericaceae)

Nicolai M. Nürk, Simon Uribe-Convers, Berit Gehrke, David C. Tank & Frank R. Blattner

The following supporting information is available for this article in additional file 1:

Phylogenetic inference and topological discordance: Figure S1. Phylogeny of Hypericaceae (detailing species names and node support). Discussion of topological discordance.

Age estimation, calibration: Discussion of fossil placement. **Figure S2**. MCC chronogram of Hypericaceae (detailing time-constraints). **Table S1**. Crown group ages of major clades, detailing results of analyses A and B.

Historical biogeography: Figure S3. Paleogeographical model designed for the stratified ancestral area reconstruction (M1). **Table S2**. Summary statistics obtained by optimization of ancestral areas over 1000 posterior trees generated by age estimations A and B detailing results using different models (M1 and M2).

Diversification rate analysis: Figure S4. Diversification rate shifts obtained by analysis A and B, with respective credible sets of distinct shift configurations. **Table S3.** Results of diversification rate analyses under the different age estimations.

Bioclimatic niche analysis: Figure S5. Niche shifts obtained by analysis A and B. **Table S3.** Results of niche analyses under the different age estimations.

Voucher: Species in this study, detailing names, reference, EMBL/Genbank ID, and species distribution (area coding and coordinates).

Phylogenetic inference

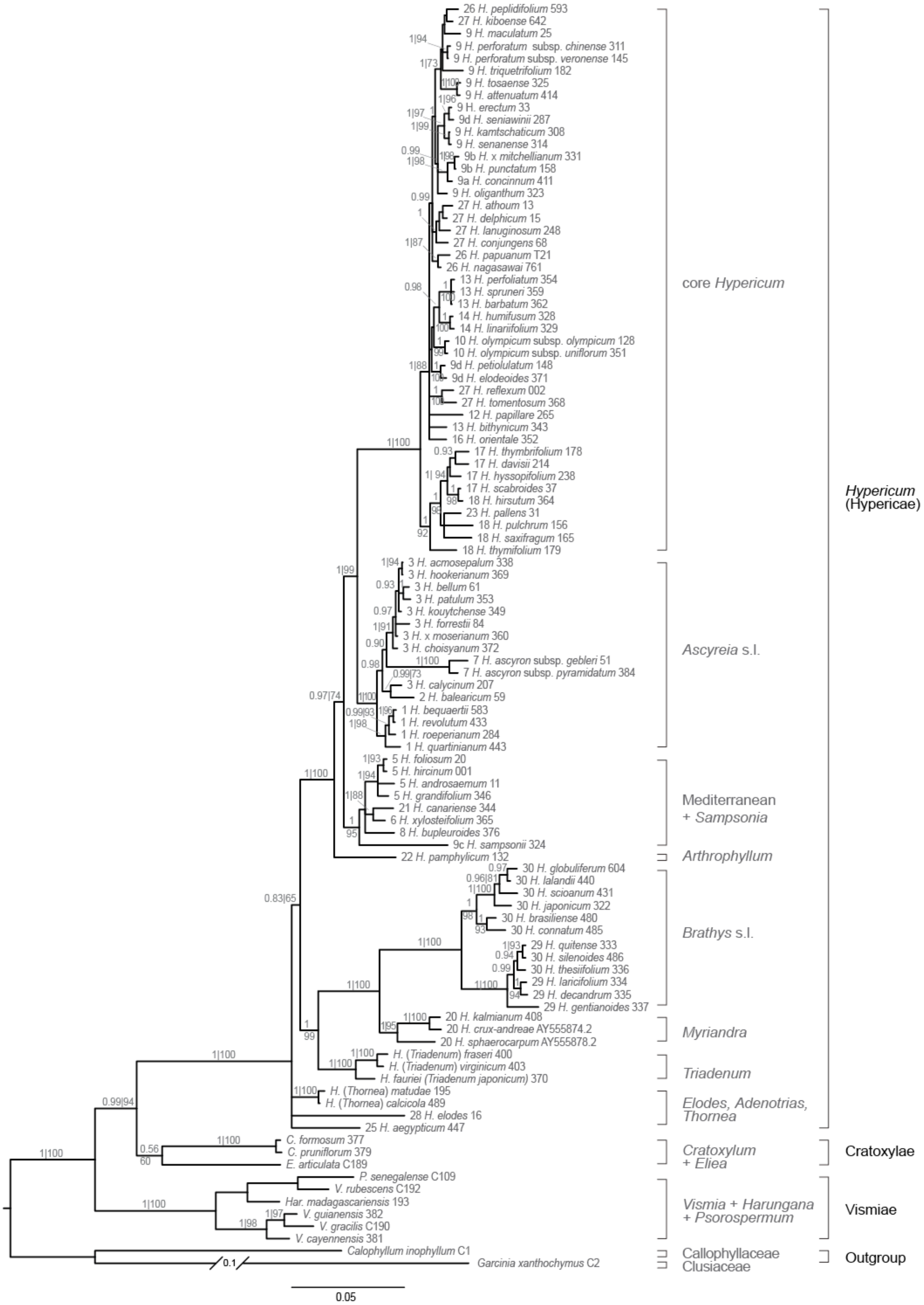

Figure S1 – Phylogeny of Hypericaceae produced from Bayesian analysis of the combined sequence data
Branch length is given in units of expected substitutions per side. Accession and clade names are detailed. Bayesian posterior probabilities and ML bootstrap support is given per branch (pp|ML; or pp above and ML below the branches). Rooting follows Xi et al. [1].

Topological discordance

Topological discordance between chloroplast (*pet*D+*trn*L–*trn*F) and nuclear (ITS) sequence inference is present only in two places, although without strong support. The first topological discordance concerns basal nodes within *Hypericum*: *H. elodes*, *H. aegypticum*, and *H. calcicola* are sister to the *Triadenum*+*Myriandra*+*Brathys* s.l. clade in the chloroplast topology, and in the rDNA tree in a grade, consisting of the earliest branchings in *Hypericum*. This topological incongruence between chloroplast and rDNA inference has also been reported in other studies (for discussion see [2]). Sánchez Meseguer et al. [3] analyzing three chloroplast marker (*psb*A-*trn*H, *trn*L-*trn*F, *trn*S-*trn*G) and ITS and using a deep sampling report no such incongruence between chloroplast and rDNA sequence inference, suggesting that the 'chloroplast' topology is a sampling issue.

The second topological incongruence has not yet been reported (this is due to missing analyses in the groups of interest using nuclear rDNA) and concerns the position of the other tribes of the Hypericaceae; Vismieae sister to *Hypericum* (Hypericeae) in the chloroplast topology (a result also shown in other studies, *e.g.*, [1]), and sister to the rest of the family in the rDNA tree. Reasons for this incongruence have not yet been investigated, and one might consider the specific mode of rDNA evolution (concerted evolution causing bidirectional homogenization) as possibly responsible processes. Moreover, Sánchez Meseguer et al. [4] studying low-copy nuclear genes (EMB2765 and PHYC) do also report discordance when compared to the chloroplast topology. Since none of the respective nodes received strong support, we concatenated the chloroplast and nuclear data set.

Age estimation: calibration

The following external calibration points were considered in the divergence time estimations, relying on six fossils which were constrained by hard minimum bounds (*i.e.* uniform calibrations), and two age estimates reported in other studies which were constrained by lognormal distributions.

1) The age of the root node (i.e. the crown node of the clusioid clade) estimated by Xi et al. [1] to 89.5 Ma (88.4–91.0 95% HPD) was constrained by a log mean of 2.4, a log standard deviation of 0.3, an offset of 78, and an initial value of 89.5.
2) The age of the crown node of the Hypericaceae estimated by Xi et al. [1] to 53.7 Ma (42.5–63.0 95% HPD) was constrained by a log mean of 2.4, a log standard deviation of 0.4, and an offset of 40.

3) Fossils seeds of *Hypericum antiguum* Balueva & V.P.Nikitin from the Upper Eocene (40.4–33.9 Ma) of West Siberia (Užaniha) [5], considered to be the oldest fossil remain of the genus [6], possess synapomorphies in the general seed morphology and the testa sculpturing with *Hypericum*. Especially, the characteristic meridional ribs on the testa resemble those of *Hypericum* sections *Elodes, Brathys*, *Trigynobrathys*, and *Sampsonia*. This fossil is certainly an extinct member of the *Hypericum* lineage. Depending on weather the fossil is a member of the crown group or the associated stem group, the age estimation will differ between the assignment to the crown node or to the stem node. Following Magallón and Sanderson [7], a fossil that possesses all apomorphies of a particular (sub)clade within the study group should be assigned to the crown node of that study group since it can be unambiguously identified as a crown group member. If the fossil presents some, but not all apomorphies of particular (sub)clades within the study group, it is assigned to the stem node of the study group. The reason is that the fossil could belong to a branch that diverged after the evolution of some apomorphies, but before the extant members of the study group diverged. In our case, assigning *H. antiguum* to the crown group of *Hypericum*, and thus assigning a time constraint to the crown node is legitimate if it is an extinct member of a certain (sub)clade within the genus. On the other hand, it is also appropriate to assign the fossil to the stem group of *Hypericum*, if the relationship to the genus but not the relationship to a certain clade within the genus is beyond doubt. Because no obvious relationship of *H. antiguum* to a certain (sub)clade within *Hypericum* can be unequivocally hypothesized, the conservative approach when using the fossil as a minimum time constraint (uniform calibration) is to assign the fossil to the *Hypericum* stem group. We designed two analyses: analysis A which uses the conservative approach and assigns the seed fossil to the stem node of *Hypericum* (*i.e.* constrains the stem node to a minimum age of 33.9 Ma) and analysis B which assigns the fossil to the crown node (*i.e.* constrains the crown node to a minimum age of 33.9 Ma) in order to enable comparison of the ages inferred in our study with the results of a recently published study [3], in which the fossil was assigned to the *Hypericum* crown node.
4) *Hypericum tertiaerum* Nikitin fossil seeds [6] from the Lower to Upper Miocene (23.0–5.3 Ma) of East Europe and Siberia were used to calibrate the stem node of *Triadenum* with a minimum age constraint of 5.3 Ma.
5) Seeds of *H. virginicum* (= *Triadenum virginicum*) from the Pleistocene [8], the earliest fossil record from North America [6], were used to calibrate the crown node of the

North American part of the *Triadenum* clade with a minimum age constraint of 0.01 Ma.

6) Pollen fossils of *Hypericum* from the Upper Pliocene (3.6–2.5 Ma) [9-11] of Andean high valleys of Colombia were used to constrain the 'Páramo' crown node (including *H. decandrum, H. laricifolium, H. quitense, H. silenoides,* and *H. thesiifolium*) to a minimum age of 2.5 Ma.
7) A macrofossil leaf assigned to *H. xylosteifolium* from the Upper Pliocene(3.6–2.5 Ma) [5] of Georgia constrained the crown node of the 'Mediterranean+*Sampsonia*' clade to a minimum age of 2.5 Ma.
8) Fossil Pliocene seeds [5], which show close affinity to the seeds of *H. perforatum*, were used to calibrate the crown node of 'core *Hypericum*' to a minimum age of 2.5 Ma.

Two approaches were conducted, which differed only in the assignment of the seed fossil *Hypericum antiguum* (see above, point 3): divergence time estimation A, which assigns the fossil to the stem node of *Hypericum*, and divergence time estimation B, which assigns the fossil to the crown node of *Hypericum*. All other calibrations remained unchanged in the two analyses. Fig. S2 gives the MCC tree produced in analysis A, and Table S1 lists the results of both analyses.

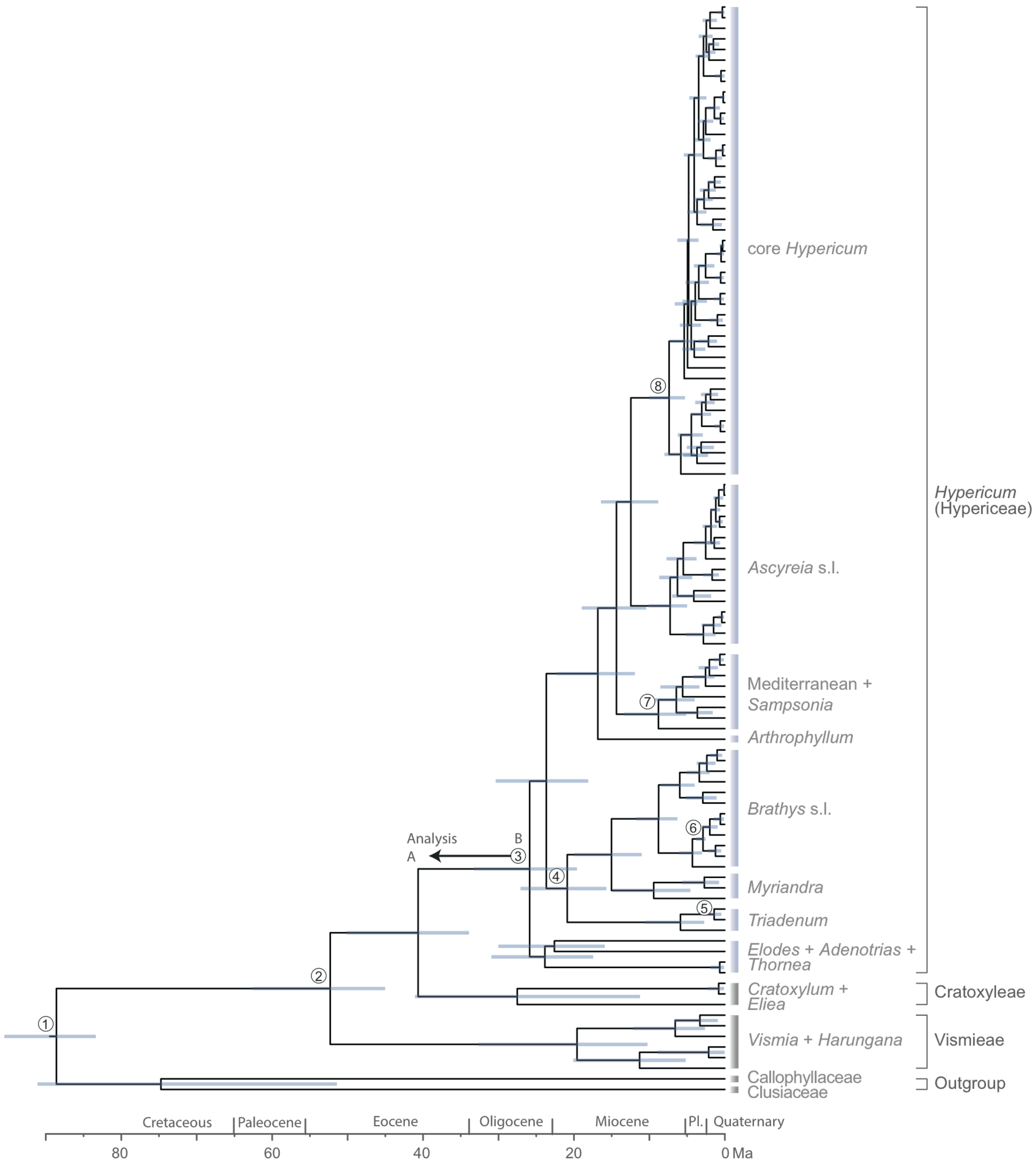

Figure S2 – Maximum clade credibility (MCC) chronogram of Hypericaceae (produced by divergence time estimation A)

Numbers in circles (1–8) indicate the assignment of external time constraints to the respective node. Node bars indicate the 95% highest posterior density (HPD). Note that in analysis A the time constraint N° 3 is assigned to the stem node of *Hypericum* (using the "including stem" option; indicated by the black arrow), whereas it is assigned to the crown node in analysis B.

Table S1 – Divergence time estimates of major clades detailing results of analyses A and B (in Ma).

Node (mrca)	Bayesian relaxed clock crown ages					
	Analysis A			Analysis B		
	mean	upper 95%HPD	lower 95%HPD	mean	upper 95%HPD	lower 95%HPD
Root	88.52	95.49	83.33	89.66	96.88	83.43
Hypericaceae	52.31	62.66	45.00	59.63	71.25	49.26
Vismieae	19.59	32.66	10.22	23.25	37.69	12.73
Hypericum + Cratoxyleae	40.65	50.17	33.91	48.85	59.48	40.69
Cratoxyleae	27.51	41.08	11.23	32.50	48.89	14.38
Hypericum	25.88	33.32	19.59	35.20	39.31	33.90
core *Hypericum* – *Brathys* s.l.	23.67	30.41	18.08	31.79	36.99	26.25
Brathys s.l. – *Triadenum*	20.89	27.10	15.71	27.74	32.89	21.95
Triadenum	5.89	10.58	2.71	7.37	13.25	3.36
Brathys s.l. + *Myriandra*	15.03	19.99	10.99	19.63	24.56	14.87
Myriandra	9.43	14.91	4.53	12.21	18.95	6.12
Brathys s.l.	8.77	11.82	6.26	11.08	14.63	7.94
core *Hypericum* – *Arthrophyllum*	16.84	22.35	11.90	21.84	28.12	16.08
core *Hypericum* – *Androsaemum*	14.37	18.97	10.40	18.38	23.65	13.56
Androsaemum + *Sampsonia*	8.80	13.43	5.10	11.16	16.72	6.63
core *Hypericum* + *Ascyreia* s.l.	12.46	16.44	8.80	15.86	20.63	11.66
Ascyreia s.l.	7.26	10.14	4.97	9.05	12.37	6.17
core *Hypericum*	7.39	10.04	5.27	9.35	12.50	6.82

Historical Biogeography

It is necessary to re-analyze the biogeography of the study group, because the parametric likelihood approach employed, *i.e.* the dispersal-extinction-cladogenesis (DEC) model [12] implemented in the program Lagrange [12, 13], takes into account the possibility of multiple states (the areas defined in the analysis) for the reconstructed distribution of ancestral populations. One of the advantages of the DEC model is its flexibility, as it can integrate temporal and paleogeographical information or species dispersal capabilities and ecological tolerance [14] by defining an instantaneous rate matrix (*Q*) of lineage dispersal between areas and extinction within an area [15]. Since there is no restriction for geographic ranges in the number of ancestral states (beside computational limitations), this approach compensates for the far distribution of several species of *Hypericum*.

a) Time slice 75–100 Ma

b) Time slice 50–75 Ma

c) Time slice 25–50 Ma

d) Time slice 0–25 Ma

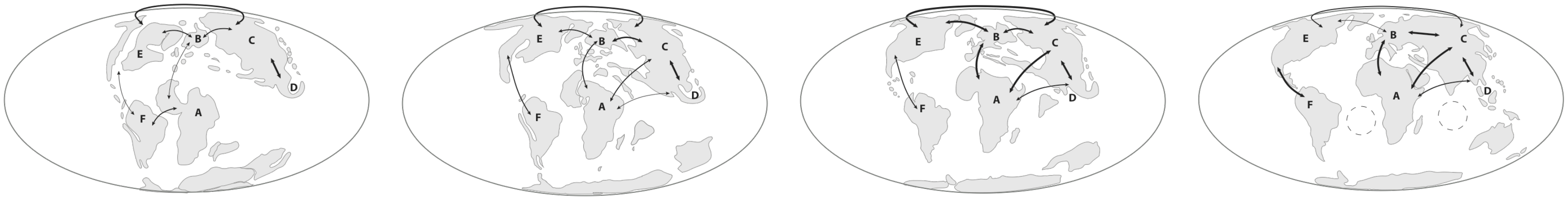

a) Time slice 75–100 Ma

	A	B	C	D	E	F
A	1	0.25	0.01	0.01	0.01	0.5
B	0.25	1	0.5	0.01	0.5	0.01
C	0.01	0.5	1	1	0.75	0.01
D	0.01	0.01	1	1	0.01	0.01
E	0.01	0.5	0.75	0.01	1	0.25
F	0.5	0.01	0.01	0.01	0.25	1

b) Time slice 50–75 Ma

	A	B	C	D	E	F
A	1	0.5	0.5	0.25	0.01	0.01
B	0.5	1	0.75	0.01	0.5	0.01
C	0.5	0.75	1	1	0.75	0.01
D	0.25	0.01	1	1	0.01	0.01
E	0.01	0.5	0.75	0.01	1	0.5
F	0.01	0.01	0.01	0.01	0.5	1

c) Time slice 25–50 Ma

	A	B	C	D	E	F
A	1	1	1	0.5	0.01	0.01
B	1	1	1	0.01	1	0.01
C	1	1	1	1	1	0.01
D	0.5	0.01	1	1	0.01	0.01
E	0.01	1	1	0.01	1	0.5
F	0.01	0.01	0.01	0.01	0.5	1

d) Time slice 0–25 Ma

	A	B	C	D	E	F
A	1	1	1	0.75	0.01	0.25
B	1	1	1	0.01	0.25	0.01
C	1	1	1	1	0.5	0.01
D	0.75	0.01	1	1	0.01	0.01
E	0.01	0.25	0.5	0.01	1	1
F	0.25	0.01	0.01	0.01	1	1

e) Connectivity through time

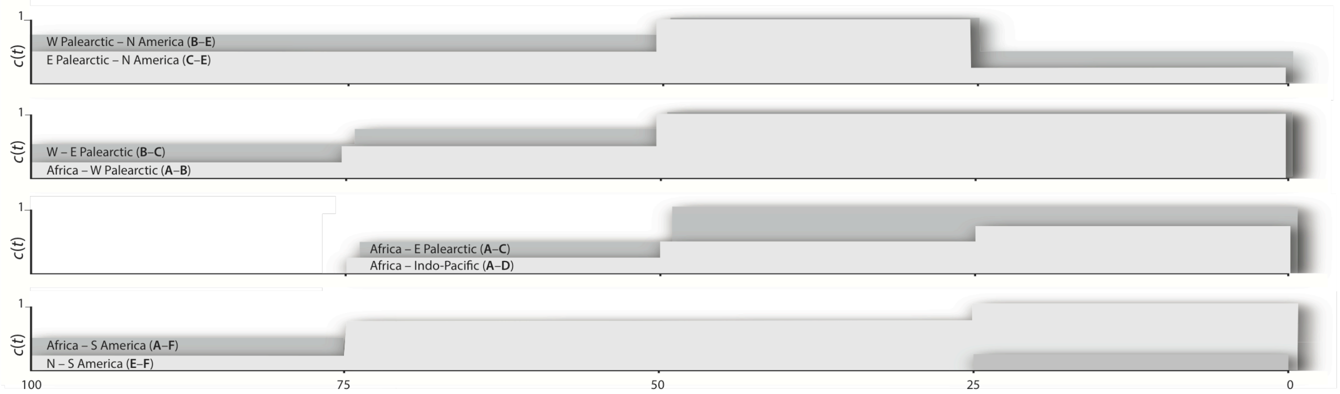

Figure S3 – Paleogeographical model designed for the stratified ancestral area optimization (M1).

Table S2. Summary statistics obtained by optimization of ancestral areas over 1000 posterior trees generated by age estimations A and B.

Node (mrca)	Analysis A (younger age estimates)						Analysis B (older age estimates)					
	Model 1 (stratified)			Model 2 (equal)			Model 1 (stratified)			Model 2 (equal)		
	area	w_i	Evidence ratio	area	w_i	Evidence ratio	area	w_i	Evidence ratio		w_i	Evidence ratio
Root	**IP**	**0.07**	**1.95**	**IP**	**0.05**	**1.31**	**IP**	**0.06**	**1.10**	**A**	**0.06**	**1.00**
	A	0.03	1.63	A	0.04	1.89	A	0.06	1.55	IP	0.06	2.32
	EP	0.02	1.70	WP	0.02	1.13	A+IP	0.04	2.46	A+IP	0.03	1.57
Hypericaceae	**A**	**0.09**	**3.60**	**A**	**0.11**	**3.97**	**A**	**0.12**	**3.28**	**A**	**0.12**	**4.19**
	A+WP	0.03	1.40	WP	0.03	1.25	A+WP	0.04	1.92	WP	0.03	1.30
	WP	0.02	1.07	IP	0.02	1.14	IP	0.02	1.44	IP	0.02	1.31
Vismieae	**A+SA**	**0.27**	**1.20**	**A+SA**	**0.33**	**1.98**	**A+SA**	**0.35**	**1.29**	**A+SA**	**0.54**	**3.41**
	A	0.22	3.70	A	0.17	2.97	A	0.27	5.02			
				SA	0.06	–						
Hypericum + Cratoxyleae	**A**	**0.06**	**1.28**	**WP**	**0.07**	**1.12**	**A+WP**	**0.10**	**1.33**	**WP**	**0.08**	**1.28**
	WP	0.04	1.12	A	0.07	1.85	A	0.07	1.22	A	0.06	1.95
	A+WP	0.04	1.82	IP	0.04	1.09	WP	0.06	2.19	A+WP	0.03	1.09
Cratoxyleae	**A+IP**	**0.26**	**1.52**	**A+IP**	**0.25**	**1.54**	**A+IP**	**0.33**	**1.98**	**A+IP**	**0.33**	**2.18**
	IP	0.17	1.08	A	0.16	1.20	A	0.17	1.21	A	0.15	1.27
	A	0.16	–	IP	0.13	–	IP	0.14	–	IP	0.12	–
Hypericum	**WP+NA**	**0.22**	**1.41**	**WP**	**0.25**	**1.11**	**WP**	**0.23**	**1.02**	**WP**	**0.25**	**1.10**
	WP	0.16	2.14	WP+NA	0.23	6.19	WP+NA	0.22	2.41	WP+NA	0.23	2.59
	WP+EP+NA	0.07	–				NA	0.09	–	NA	0.09	–
core *Hypericum* – *Brathys* s.l.	**WP+NA**	**0.32**	**2.63**	**WP+NA**	**0.34**	**1.84**	**WP+NA**	**0.37**	**1.97**	**WP+NA**	**0.37**	**1.91**
	WP	0.12	2.02	WP	0.19	2.17	WP	0.19	2.17	WP	0.19	2.27
	NA	0.06	1.19	NA	0.09	–	NA	0.09	–	NA	0.08	–
Brathys s.l. – *Triadenum*	**NA**	**0.51**	**6.35**	**NA**	**0.49**	**5.03**	**NA**	**0.51**	**4.49**	**NA**	**0.50**	**5.26**
Triadenum	**EP+NA**	**0.78**	**7.97**	**EP+NA**	**0.77**	**9.30**	**EP+NA**	**0.80**	**10.53**	**EP+NA**	**0.80**	**11.77**
Brathys s.l. + *Myriandra*	**NA**	**0.50**	**3.61**	**NA**	**0.49**	**5.12**	**NA**	**0.52**	**4.49**	**NA**	**0.48**	**4.54**
Myriandra	**NA**	**0.98**	**163.03**	**NA**	**0.97**	**161.56**	**NA**	**0.98**	**109.85**	**NA**	**0.98**	**185.02**
Brathys s.l.	**NA+SA**	**0.33**	**5.23**	**NA+SA**	**0.29**	**5.32**	**NA+SA**	**0.33**	**4.61**	**NA+SA**	**0.28**	**4.04**
core *Hypericum* – *Arthrophyllum*	**WP**	**0.68**	**7.21**	**WP**	**0.73**	**11.03**	**WP**	**0.68**	**7.79**	**WP**	**0.72**	**10.42**
core *Hypericum* – *Androsaemum*	**WP**	**0.61**	**5.34**	**WP**	**0.67**	**6.03**	**WP**	**0.60**	**5.12**	**WP**	**0.65**	**5.31**
Androsaemum + *Sampsonia*	**WP**	**0.63**	**2.27**	**WP**	**0.53**	**1.38**	**WP**	**0.62**	**2.10**	**WP**	**0.50**	**1.21**
	WP+EP	0.28	9.06	WP+EP	0.38	12.04	WP+EP	0.30	9.40	WP+EP	0.41	11.92
core *Hypericum* + *Ascyreia* s.l.	**WP**	**0.57**	**7.55**	**WP**	**0.65**	**14.25**	**WP**	**0.56**	**7.45**	**WP**	**0.63**	**13.19**
Ascyreia s.l.	**A+WP+EP**	**0.30**	**1.91**	**A+WP+IP**	**0.22**	**1.90**	**A+WP+EP**	**0.31**	**1.86**	**A+WP+IP**	**0.22**	**1.74**
	A+WP+EP+IP	0.16	3.79	A+WP+EP+IP	0.11	2.11	A+WP+EP+IP	0.17	3.42	A+WP+EP+IP	0.13	2.31
				A+WP+EP	0.05	–				A+WP+EP+IP+NA	0.05	–
core *Hypericum*	**WP**	**0.60**	**2.25**	**WP**	**0.69**	**3.48**	**WP**	**0.59**	**2.49**	**WP**	**0.68**	**3.50**
	WP+EP	0.27	6.27				WP+EP	0.24	5.25			

Bold font indicates the most favored biogeographic scenario per node reconstructed under the different approaches as indicated by composite Akaike weights (w_i) and evidence ratios (w_i/w_j). Up to three alternative scenarios are given unless $w_i \geq 0.2$, or $w_i/w_j \geq 3$.
A, Afrotropical; **WP**, western Palearctic; **EP**, eastern Palearctic; **IP**, Indo-Pacific; **NA**, North America (Nearctic); **SA**, South America (Neotropic).

Diversification Rate Analyses

The summary across the entire posterior produced in analysis A and B is given in Fig. S4, together with the credible set of distinct shift configurations that accounts for >87% of the probability of the data. The 95% credibility set contained six (A) and five (B) distinct shift configurations, but all with a posterior probability of a magnitude smaller than the third-probable shift configuration.

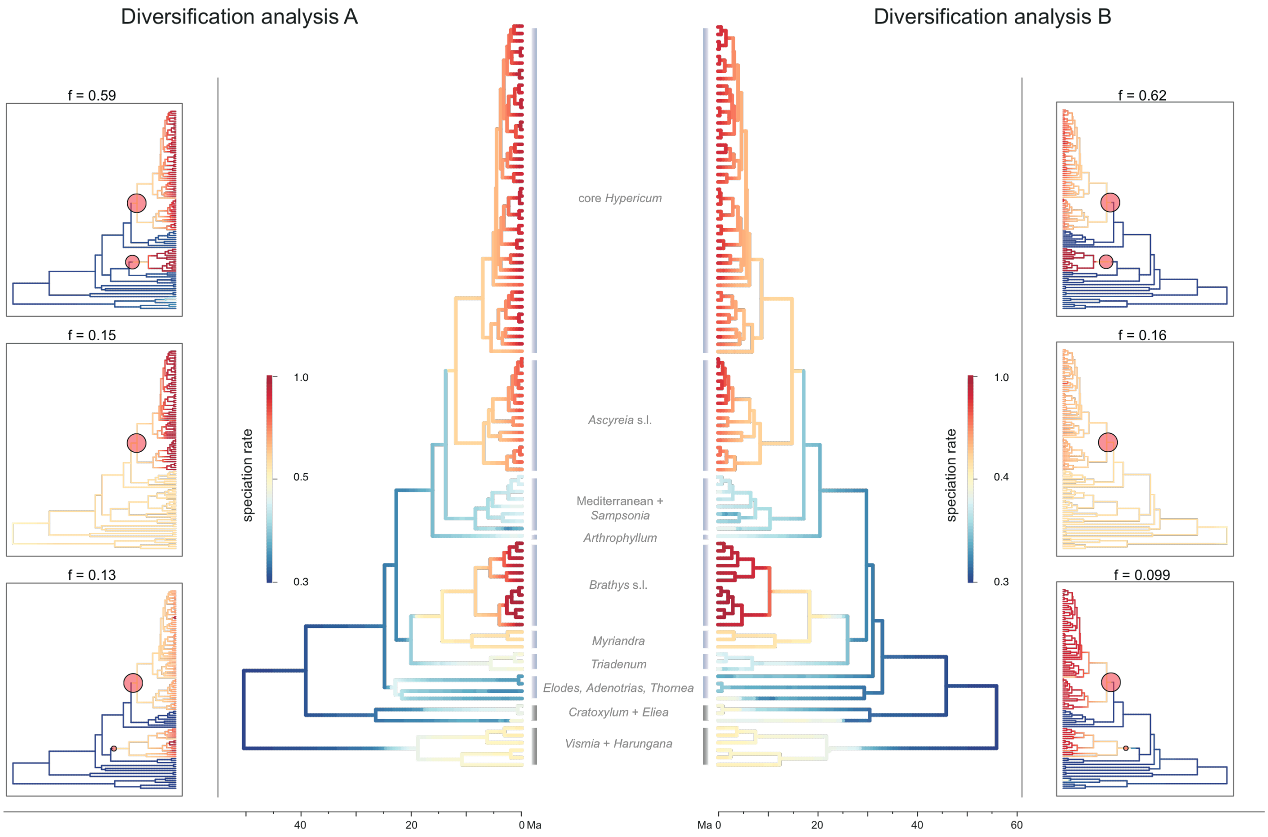

Figure S4 – Diversification rate shifts obtained by analysis A and B
The mean 'phylorate' plots are given in the center detailing speciation rates. The first three shift configurations from the Bayesian credible sets of distinct shift configurations accounting for ≥87% of the posterior probability are given to the left (A) and to the right (B), respectively. In the figure top left/right the maximum a posterior configuration is given. Circle sizes indicate marginal probabilities of the respective rate shift (f = frequency = posterior probability).

Bioclimatic niche analysis

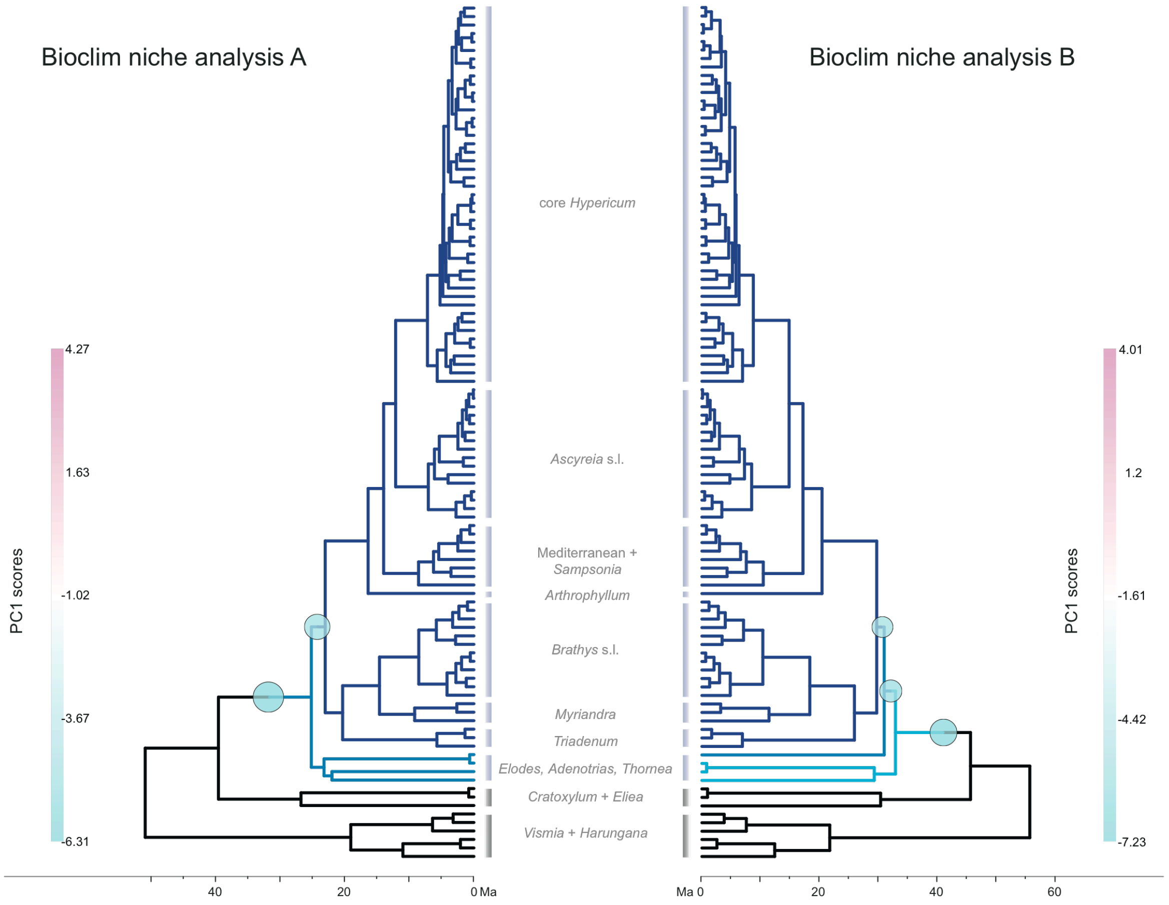

Figure S5. Niche shifts obtained by analysis A and B
Circles on the trees locate shifts, and circle colors corresponding to the scales indicate the magnitude of shifts. Circle size indicates posterior probabilities (pp) of shifts. Arbitrarily chosen colors of branches highlight clades, which were estimated to possess distinctive adaptive optima (cutoff ≥0.2 pp).

Table S3 - Results of diversification rate and bioclimatic niche analyses under different age estimations (analysis A and B).

Node (mrca)	Diversification rates				Bioclim niche shifts			
	Analysis A		Analysis B		Analysis A		Analysis B	
	shift probability	speciation rate	shift probability	speciation rate	pp	phenotypic optimum	pp	phenotypic optimum
Hypericaceae	—	0.66	—	0.54	—	0.00	—	0.00
		(0.46–0.88)		(0.38–0.75)				
Vismieae	—	0.43	—	0.37	—	1.63	—	1.96
		(0.19–0.83)		(0.17–0.73)		(0.07)		(0.11)
Cratoxyleae	—	0.41	—	0.36	—	1.11	—	1.00
		(0.18–0.81)		(0.15–0.68)		(0.15)		(0.19)
Hypericum	—	0.75	—	0.60	0.53	-4.24	0.41	-4.66
		(0.54–1.0)		(0.44–0.82)		(0.01)		(0.01)
core *Hypericum* – *Brathys* s.l.	—	0.83	—	0.66	0.34	-4.40	0.26	-4.78
		(0.59–1.08)		(0.49–0.87)		(0.01)		(0.01)
Brathys s.l.	0.64	1.04	0.63	0.89	—	-3.67	—	-3.88
		(0.54–1.75)		(0.46–1.53)		(0.06)		(0.07)
core *Hypericum* + *Ascyreia* s.l.	0.93	1.06	0.93	0.83	—	-4.67	—	-5.15
		(0.75–1.51)		(0.56–1.19)		(0.03)		(0.04)
core *Hypericum*	—	1.07	—	0.84	—	-5.09	—	-5.72
		(0.75–1.52)		(0.56–1.20)		-0.03		-0.03

For the diversification rate analysis, detected shifts are marked by their probability. The mean speciation rate (species/Ma) per clade is detailed with the 5%, and 95% highest posterior density in brackets below. For the bioclimatic niche analysis, shifts are marked by their probability, and the new phenotypic optimum (PC1 score optimum) is detailed with the standard error in brackets below.

Voucher

Species in this study, detailing names, reference (collector, col. number, and herbarium acronym; for species new in this study), EMBL/Genbank ID (ITS, *pet*D, *trn*L-*trn*F), species distribution (area code used in the ancestral area estimation: A, Afrotropical [central Africa, the southern Arabian peninsula, Madagascar, and the West Indian Ocean islands]; WP, western Palearctic; EP, eastern Palearctic; IP, Indo-Pacific [SE tropical Asia, Australasia, and the Pacific]; NA, North America [Nearctic]; SA, South America [Neotropic]), and collection sites (latitude, longitude).

Calophyllum inophyllum L., AB110820, FM178053, –, A+IP; ***Cratoxylum formosum*** (Jack) Benth. & Hook.f. ex Dyer subsp. *formosum,* HE653674, –, –, IP, 7.4167, 99.5833; ***Cr. pruniflorum*** Dyer, Larson, Larson, Nielsen & Santisuk 32141 (B), HE653675, LK871665, –, IP, 12.8667, 102.1667; ***Eliea articulata*** Cambess., KC709409, –, –, A, -13.6500, 48.6700; ***Garcinia xanthochymus*** Hook.f. ex T.Anderson, AB110817, FM178059, –, IP; ***Harungana madagascariensis*** Poir., A.J.M. Leeuwenberg 8143 (B), HE653670, –, LK871744, A, 4.5800, 9.5300; ***Hypericum acmosepalum*** N.Robson, N.M. Nürk 401 (GAT), HE653396, LK871666, –, EP+IP, 25.0100, 102.7000; ***H. aegypticum*** L., Turland 111 (BM), HE653403, LK871667, LK871745, WP, 30.7436, -9.2603; ***H. androsaemum*** L., C. Scheriau HEID808382 (HEID), HE653406, LK871668, LK871746, WP+EP, 42.2430, 12.2210; ***H. ascyron*** L. subsp. *gebleri* (Ledeb.) N.Robson, Wan & Chow 81093 (BM), HE653410, LK871669, –, EP+IP, 43.2181, 141.6694; ***H. ascyron*** L. subsp. *pyramidatum* (Aiton) N.Robson, Ames 28349 (ISU), LK871650, LK871670, LK871747, NA, 42.0400, -93.6400; ***H. athoum*** Boiss. & Orph., C. Scheriau HEID801636 (HEID), HE653413, LK871671, LK871748, WP, 40.7330, 24.6670; ***H. attenuatum*** Fisch. ex Choisy, F.R. Blattner FB2010/032a (GAT), HE662752, –, –, EP, 47.0171, 119.6254; ***H. balearicum*** L., M.J. Cannon 3780 (BM), HE653418, LK871672, –, WP, 39.7400, 3.3100; ***H. barbatum***

Jacq., N.M. Nürk 410 (GAT), HE653420, LK871673, –, WP, 41.1500, 22.2200; ***H. bellum*** H.L.Li, SBEC 0424 (BM), HE653425, LK871674, –, IP, 29.9906, 95.3197; ***H. bequaertii*** De Wild., B. Gehrke BG367 (Z), LK871651, LK871675, LK871749, A, 0.3501, 29.9246; ***H. bithynicum*** Boiss., N.M. Nürk 398 (GAT), HE653427, LK871676, –, EP, 41.7500, 42.6500; ***H. brasiliense*** Choisy, Gentry & Solomon 44755 (BM), HG004770, –, LK871750, SA, -16.1000, -68.0667; ***H. bupleuroides*** Griseb., A. Gröger & W. Lobin 113-3 (M), HE653429, LK871677, LK871751, WP+EP, 41.6400, 42.5800; ***H. calcicola*** Standl. & Steyerm., D.E. Breedlove & R.F. Thorne 21104 (BM), LK871652, LK871678, LK871752, NA, 16.1200, -91.7000; ***H. calycinum*** L., D. McClintock s.n. [1993] (BM), HE653430, LK871679, LK871753, WP, 41.1700, 29.0000; ***H. canariense*** L., N.M. Nürk 386 (GAT), HE653436, LK871680, –, WP, 28.3500, -16.7500; ***H. choisyanum*** Wall. ex N.Robson, Ikeda et al. 20913019 (TI), HE653441, LK871681, –, IP, 28.1611, 85.4322; ***H. concinnum*** Benth., D. Potter & P. Lash 110612-01 (GAT), HE653442, –, –, NA, 38.7236, -120.9592; ***H. conjungens*** N.Robson, L.B. Mwasumbi 16191A (BM), HE653444, LK871682, –, A, -8.9667, 33.6500; ***H. connatum*** Lam., M. Serrano et al. 6893 (BM), HG004774, LK871683, LK871754, SA, -20.3294, -64.0422; ***H. crux-andreae*** (L.) Crantz, AY555874.2, –, –, NA, 30.2900, -84.8300; ***H. davisii*** N.Robson, A. Çubukçu s.n. [1978] (BM), HE653448, LK871684, –, EP, 39.9316, 45.3031; ***H. decandrum*** Turcz., M. Weigend 9102 (B), HE653449, LK871685, –, SA, -2.7800, -79.2200; ***H. delphicum*** Boiss. & Heldr., C. Scheriau HEID808395 (HEID), HE653451, LK871686, LK871755, WP, 38.0670, 24.4670; ***H. elodeoides*** Choisy, Ikeda et al. 20911111 (TI), HE653458, LK871687, –, EP+IP, 27.6952, 86.7398; ***H. elodes*** L., C. Scheriau HEID808396 (HEID), HE653460, LK871688, LK871756, WP, 48.6980, -0.3680; ***H. erectum*** Thunb., N.M. Nürk 383 (BM), HE653467, LK871689, LK871757, EP, 43.6492, 142.7911; ***H. fauriei*** R.Keller, N.M. Nürk 414 (GAT), HE653665, LK871690, LK871758, EP, 36.7186, 138.4936; ***H. foliosum*** Aiton, H. Schaefer HS208 (K), HE653473, LK871691, –, WP, 38.5800, -28.7100; ***H. forrestii*** (Chitt.) N.Robson, SBEC 0472 (BM), HE653474, LK871692, –, EP+IP, 27.8200, 99.7100; ***H. fraseri*** Steud., S.R. Hill 17290 (GH), HE653663, LK871693, –, NA, 47.8000, -91.0833; ***H. gentianoides*** (L.) Britton, Sterns & Poggenb., N.M. Nürk 384 (GAT), HE653479, LK871694, –, NA, 44.7075, -63.8913; ***H. globuliferum*** R.Keller, B. Gehrke BG246 (Z), LK871653, –, LK871759, A, -19.7670, 48.3120; ***H. grandifolium*** Choisy, N.M. Nürk 403 (GAT), HE653487, LK871695, –, WP, 28.5300, 16.2800; ***H. hircinum*** L., C. Scheriau HEID808404 (HEID), LK871654, LK871696, LK871760, WP, 42.3060, 9.1510; ***H. hirsutum*** L., N.M. Nürk 390 (GAT), HE653498, LK871697, –, WP+EP, 49.0833, 20.3833; ***H. hookerianum*** Wight & Arn., N.M. Nürk 413 (GAT), HE653503, LK871698, LK871761, EP+IP, 27.4500, 90.1667; ***H. humifusum*** L., N.M. Nürk 381 (GAT), HE653509, LK871699, LK871762, WP, 35.2515, -5.4239; ***H. hyssopifolium*** Chaix, P. Bamps 9004 (BM), HE653511, LK871700, –, WP, 40.1000, -1.1000; ***H. japonicum*** Thunb., K. Masuda 3360 (KYO), HE653512, LK871701, LK871763, EP+IP, 24.2139, 121.3389; ***H. kalmianum*** L., N.M. Nürk 397 (GAT), HG004780, LK871702, LK871764, NA, 42.2702, -81.8613; ***H. kamtschaticum*** Ledeb., N.M. Nürk 366 (GAT), HE653516, LK871703, –, EP, 43.6536, 142.8186; ***H. kiboense*** Oliv., B. Gehrke BG149C (Z), LK871655, LK871704, LK871765, A, 1.2200, 35.4800; ***H. kouytchense*** H.Lév., N.M. Nürk 431 (GAT), HE653520, LK871705, –, IP, 27.7000, 103.2500; ***H. lalandii*** Choisy, B. Gehrke BG207 (Z), LK871656, LK871706, LK871766, A, -24.7000, 25.9000; ***H. lanuginosum*** Lam., R. Ulrich s.n. [1998] (BM), HE653525, LK871707, –, WP, 36.8800, 34.5800; ***H. laricifolium*** Juss., M. Weigend 910 (B), HE653526, LK871708, LK871767, SA, 5.5575, -73.1194; ***H. linariifolium*** Vahl, N.M. Nürk 379 (GAT), HE653532, LK871709, LK871768, WP, 41.7400, -8.5100; ***H. maculatum*** Crantz, C. Scheriau HEID808356 (HEID), LK871657, LK871710, LK871769, WP, 50.9300, 14.2700; ***H. matudae*** Lundell, D.E. Breedlove 40408 (B), HE653661, –, –

, NA, 13.5667, -85.7000; ***H. x mitchellianum*** Rydb., N.M. Nürk 382 (GAT), HE653541, LK871711, LK871770, NA, 36.1000, -82.1300; ***H. x moserianum*** Luquet ex André, N.M. Nürk 409 (GAT), HE653551, LK871712, –, EP, 50.9200, 5.8900; ***H. nagasawai*** Hayata, C.-K. Liou et al. 821 (PE), LK871658, LK871713, LK871771, IP, 23.4686, 120.9608; ***H. oliganthum*** Franch. & Sav., T. Sawada 236 (KYO), HE653559, LK871714, LK871772, EP, 35.7800, 139.6900; ***H. olympicum*** L. forma *olympicum,* W. Greuter 16146 (BM), HE653562, LK871715, –, WP, 41.2000, 23.7667; ***H. olympicum*** L. forma *uniflorum* D.Jord. & Kozuharov, N.M. Nürk 387 (GAT), HE653564, LK871716, –, WP, 40.9200, 24.1000; ***H. orientale*** L., N.M. Nürk 396 (GAT), HE653565, LK871717, –, WP+EP, 42.5600, 44.7600; ***H. pallens*** Banks & Sol., C. Scheriau HEID801626 (HEID), HE653567, LK871718, –, WP, 37.0190, 34.2890; ***H. pamphylicum*** N.Robson & P.H.Davis, R. Ulrich s.n. [1998] (BM), HE653569, –, LK871773, WP, 36.8217, 31.6914; ***H. papillare*** Boiss. & Heldr., A. Çubukçu & A. Basaran A-12 (BM), HE653570, LK871719, –, WP, 37.8836, 32.3068; ***H. papuanum*** Ridl., HE653571, –, –, IP, -4.6615, 140.0970; ***H. patulum*** Thunb., N.M. Nürk 582 (GAT), LK871659, LK871720, –, EP, 26.0200, 98.6300; ***H. peplidifolium*** A.Rich., B. Gehrke BG078 (Z), LK871660, LK871721, LK871774, A, -8.8333, 33.3333; ***H. perfoliatum*** L., N.M. Nürk 385 (GAT), HE653576, LK871722, –, WP, 35.0000, -6.1167; ***H. perforatum*** L. subsp. *chinense,* N.M. Nürk 353 (GAT), HE653577, LK871723, LK871775, EP+IP, 36.7394, 139.5017; ***H. perforatum*** L. subsp. *veronense,* S.L. Jury and M. Ait Lafkih & B. Tahiri 14865 (BM), LK871661, LK871724, LK871776, WP, 34.0667, -5.5500; ***H. petiolulatum*** Hook.f. & Thomson ex Dyer, A.J.C. Grierson & D.G. Long 2549 (BM), HE653582, LK871725, –, EP+IP, 27.3500, 91.0167; ***H. pulchrum*** L., Cubr 39891 (B), HE653599, –, –, WP, 47.7833, 6.4167; ***H. punctatum*** Lam., D.E. Boufford & E.W. Wood 23250 (BM), HE653601, –, –, NA, 38.1250, -90.6750; ***H. quartinianum*** A.Rich., B. Gehrke BG273 (Z), LK871662, LK871726, –, A, 8.8500, 33.2833; ***H. quitense*** R.Keller, M. Weigend 9100 (B), HE653606, LK871727, –, SA, -2.6500, -78.7167; ***H. reflexum*** L., F. Blattner FB2008/004 (GAT), HE653607, LK871728, LK871777, WP, 28.5578, -16.2500; ***H. revolutum*** Vahl, B. Gehrke BG100 (Z), LK871663, LK871729, LK871778, A, 6.9100, 40.1800; ***H. roeperianum*** G.W.Schimp. ex A.Rich., W.T. Stearn s.n. [1977] (BM), HE653614, –, –, A, 13.2000, 37.8800; ***H. sampsonii*** Hance, NN 16917 (KYO), HE653620, LK871730, –, EP, 27.6778, 117.9658; ***H. saxifragum*** N.Robson & Hub.-Mor., R. Ulrich s.n. [6.10.1997] (BM), HE653621, –, –, WP, 37.0441, 30.1279; ***H. scabroides*** N.Robson & Poulter, C. Scheriau HEID808412 (HEID), HE653623, LK871731, –, WP+EP, 47.1850, 4.9520; ***H. scioanum*** Chiov., B. Gehrke BG081 (Z), LK871664, LK871732, LK871779, A, 9.0500, 38.7333; ***H. senanense*** Maxim., N.M. Nürk 361 (GAT), HE653629, LK871733, –, EP, 43.6489, 142.7992; ***H. seniawinii*** Maxim., X. Bai-Zhong 3778 (BM), HE653630, –, –, EP+IP, 24.9445, 112.9114; ***H. silenoides*** Juss., J. C. Solomon 16431 (BM), HG004762, –, LK871780, SA, -16.1333, -68.1167; ***H. sphaerocarpum*** Michx., AY555878.2, –, –, NA, 35.2095, -86.7441; ***H. spruneri*** Boiss., N.M. Nürk 408 (GAT), HE653632, LK871734, –, WP, 41.1700, 20.1900; ***H. thesiifolium*** Kunth, M. Weigend 9119 (B), HE653644, LK871735, –, SA, 15.3814, -90.1858; ***H. thymbrifolium*** Boiss. & Noë, A. Çubukçu 3 (BM), HE653645, LK871736, –, WP+EP, 39.2606, 37.2415; ***H. thymifolium*** Banks & Sol., R. Ulrich 0/52 (BM), HE653646, LK871737, –, WP, 33.8800, 35.5000; ***H. tomentosum*** L., N.M. Nürk 412 (GAT), HE653649, LK871738, –, WP, 35.0333, -4.1667; ***H. tosaense*** Makino, T. Kobayashi 41978 (KYO), HE653650, LK871739, LK871781, EP, 34.8333, 134.9000; ***H. triquetrifolium*** Turra, J,R. Akeroyd, S.L. Jury & F.J.Rumsey 3572 (BM), HE653651, LK871740, LK871782, WP, 37.9167, 15.3333; ***H. virginicum*** L., R.S. Mitchel & J. Focht 8507 (GH), HE653667, LK871741, –, NA, 41.5051, -71.5675; ***H. xylosteifolium*** (Spach) N.Robson, N.M. Nürk 411 (GAT), HE653438, LK871742, –, WP+EP, 41.3890, 41.4240; ***Psorospermum senegalense*** Spach, –, –,

KC709222, A, 6.7700, 11.9500; ***Vismia cayennensis*** (Jacq.) Pers., Mori, Lokova & Keeley 25662 (B), HE653671, –, –, SA, 3.9833, -52.5667; ***V. gracilis*** Hieron., KC709410, –, KC709112, SA, 1.8300, -59.0800; ***V. guianensis*** (Aubl.) Choisy, Jansen-Jacobs, Lilwah, Raghoenandan, Scheplitz & Vermeer 5501 (B), HE653672, LK871743, –, SA, 2.0000, -59.5667; ***V. rubescens*** Oliv., KC709411, –, –, A, 0.6100, 10.4000.

References (Supplements)

1. Xi Z, Ruhfel BR, Schaefer H, Amorim AM, Sugumaran M, Wurdack KJ, Endress PK, Matthews ML, Stevens PF, Mathews S, Davis CC: **Phylogenomics and a posteriori data partitioning resolve the Cretaceous angiosperm radiation Malpighiales.** *Proc Natl Acad Sci U S A* 2012, **109:**17519-17524.
2. Nürk NM, Madriñán S, Carine MA, Chase MW, Blattner FR: **Molecular phylogenetics and morphological evolution of St. John's wort (*Hypericum*; Hypericaceae).** *Mol Phylogenet Evol* 2013, **66:**1-16.
3. Sanchez Meseguer A, Aldasoro JJ, Sanmartín I: **Bayesian inference of phylogeny, morphology and range evolution reveals a complex evolutionary history in St. John's wort (*Hypericum*).** *Mol Phylogenet Evol* 2013, **67:**379-403.
4. Meseguer AS, Sanmartín I, Marcussen T, Pfeil BE: **Utility of low-copy nuclear markers in phylogenetic reconstruction of *Hypericum* L. (Hypericaceae).** *Plant Syst Evol* 2014, **300:**1503-1514.
5. Arbuzova O: **Hypericum.** In *Iskopaemye tsvetkovye rastenija Rossii i sopredel'nyh gosudarstv [Fossil flowering plants of Russia and adjacent countries].* Vol IV, Nyctaginaceae-Salicaceae. Edited by Budantsev L. Saint Petersburg; 2005: 48-52
6. Sanchez Meseguer A, Sanmartín I: **Paleobiology of the genus *Hypericum* (Hypericaceae): a survey of the fossil record and its palaeogeographic implications.** *An Jard Bot Madr* 2012, **69:**97-106.
7. Magallón SS, Sanderson MJ: **Absolute diversification rates in Angiosperm clades.** *Evolution* 2001, **55:**1762-1780.
8. Miller NJ, Calkin PE: **Paleoecological interpretation and age of an interstadial lake bed in western New York.** *Quat Res* 1992, **37:**75-88.
9. Van der Hammen T, Werner JH, Van Dommelen H: **Palynological record of the upheaval of the northern Andes: a study of the Pliocene and Lower Quaternary of the Colombian eastern Cordillera and the early evolution of its high-Andean biota.** *Rev Palaeobot Palynol* 1973, **16:**1-122.
10. Wijninga VM: **Neogene ecology of the Salto de Tequendama site (2475 m altitude, Cordillera Oriental, Colombia): the paleobotanical record of montane and lowland forests.** *Rev Palaeobot Palynol* 1996, **92:**97-156.
11. Wijninga VM, Kuhry P: **A Pliocene flora from the Subachoque Valley (Cordillera Oriental, Colombia).** *Rev Palaeobot Palynol* 1990, **62:**249-254.

12. Ree RH, Moore BR, Webb CO, Donoghue MJ: **A likelihood framework for inferring the evolution of geographic range on phylogenetic trees.** *Evolution* 2005, **59:**2299-2311.
13. Ree RH, Smith SA: **Maximum likelihood inference of geographic range evolution by dispersal, local extinction, and cladogenesis.** *Syst Biol* 2008, **57:**4-14.
14. Ree RH, Sanmartín I: **Prospects and challenges for parametric models in historical biogeographical inference.** *J Biogeogr* 2009, **36:**1211-1220.
15. Buerki S, Forest F, Alvarez N, Nylander JAA, Arrigo N, Sanmartín I: **An evaluation of new parsimony-based versus parametric inference methods in biogeography: a case study using the globally distributed plant family Sapindaceae.** *J Biogeogr* 2011, **38:**531-550.
